# *Neospora caninum* infection induces an isolate virulence-dependent pro-inflammatory gene expression profile in bovine monocyte-derived macrophages

**DOI:** 10.1186/s13071-020-04239-3

**Published:** 2020-07-25

**Authors:** Marta García-Sánchez, Laura Jiménez-Pelayo, Pilar Horcajo, Esther Collantes-Fernández, Luis Miguel Ortega-Mora, Javier Regidor-Cerrillo

**Affiliations:** 1 Saluvet, Animal Health Department, Faculty of Veterinary Sciences, Complutense University of Madrid, Ciudad Universitaria s/n, 28040 Madrid, Spain; 2 Saluvet-Innova, Faculty of Veterinary Sciences, Complutense University of Madrid, Ciudad Universitaria s/n, 28040 Madrid, Spain

**Keywords:** *Neospora caninum*, Bovine macrophages, Isolate virulence, Transcriptome, Innate immune response, Host-parasite interactions

## Abstract

**Background:**

*Neospora caninum* is an obligate intracellular parasite, and its ability to survive inside host immune cells may be a key mechanism for the establishment of infection in cattle. *In vitro* studies carried out by our group have shown that *N. caninum* is able to replicate in bovine macrophages (MØs), alter their microbicidal mechanisms and exploit their motility. Furthermore, host-cell control seems to be isolate virulence-dependent.

**Methods:**

To investigate the molecular basis underlying the innate responses in MØs against *N. caninum* and the mechanisms of parasite manipulation of the host cell environment, the transcriptome profile of bovine monocyte-derived MØs infected with high-virulence (Nc-Spain7) or low-virulence (Nc-Spain1H) *N. caninum* isolates was studied.

**Results:**

Functional enrichment revealed upregulation of genes involved in chemokine signalling, inflammation, cell survival, and inhibition of genes related with metabolism and phagolysosome formation. MØs activation was characterized by the induction of a predominantly M1 phenotype with expression of *TLR2*, *TLR3* and *TLR9* and activation of the NF-ƙB signalling pathway. Heat-killed *N. caninum* tachyzoites failed to activate NF-ƙB, and to inhibit lysosomal activity and apoptosis, which indicates active modulation by the parasite. The FoxO signalling pathway, Th1-Th2 differentiation, glycosaminoglycan degradation and apoptosis were pathways enriched only for low virulent Nc-Spain1H infection. In addition, Nc-Spain1H infection upregulated the *IL12A* and *IL8* pro-inflammatory cytokines, whereas *IL23* was downregulated by high virulent Nc-Spain7.

**Conclusions:**

This study revealed mechanisms implicated in the recognition of *N. caninum* by bovine MØs and in the development of the subsequent immune response. NF-ƙB seems to be the main signalling pathway implicated in the pro-inflammatory bovine MØs response against this pathogen. Apoptosis and phagolysosome maturation are processes repressed by *N. caninum* infection, which may guarantee its intracellular survival. The results also indicate that Nc-Spain7 may be able to partially circumvent the pro-inflammatory response whereas Nc-Spain1H induces a protective response to infection, which may explain the more efficient transmission of the high-virulence Nc-Spain7 isolate observed *in vivo*.
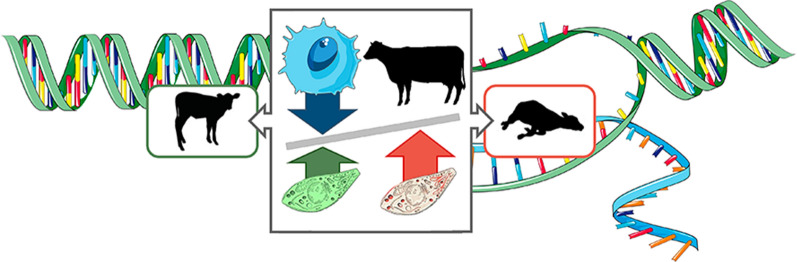

## Background

*Neospora caninum* is an apicomplexan parasite that is phylogenetically related to *Toxoplasma gondii* and is responsible for major economic losses due to reproductive failure in cattle [1]. Macrophages (MØs) are key effectors in the innate immune system and play a major role in early host resistance to *N. caninum* infection [2]. MØs are able to detect pathogens by means of pattern recognition receptors (PRRs), resulting in phagocytosis and elimination of the pathogen by nitrogen intermediates, reactive oxygen species (ROS) and lysosomal enzymes. These cells also link the innate and the adaptive response by the release of cytokines and chemokines and by their ability to present antigens to naïve T-cells [3, 4].

Studies in murine MØs have identified the TLR2-MAPK, TLR3-TRIF and TLR11-MEK/ERK pathways and NLRP3-inflammasome activation as signalling pathways implicated in host resistance against *N. caninum*, that trigger the production of pro-inflammatory cytokines [5–8]. Several studies have demonstrated that *N. caninum* has evolved mechanisms to evade the immune response mounted by murine MØs. Enhanced expression of C-type lectin receptor Dectin-1 has been related to the downregulation of ROS [9], p38 MAPK-dependent GPCR/PI3K/AKT pathway activation with the downregulation of IL-12 production [10], and upregulation of *PPARG* receptor and inhibition of NF-ƙB activation with the polarization of MØs from a M1 (pro-inflammatory) to a M2 (anti-inflammatory) phenotype [11]. In human MØs, MEK 1/2-mediated expression of cathelicidins has been proposed as a mechanism of defence against *N. caninum* infection [12]. Although these studies provide important advancements towards the understanding of the *N. caninum*-MØ interaction, it is important to consider that mice and humans are not natural hosts for *N. caninum* and that essential differences exist regarding the immune response in cattle, the main natural host for *N. caninum*. These include the lack of *TLR11* and *TLR12* in the genomes of cattle, which are by contrast present in mice, or the limited (65–77% in average) nucleotide homology to human TLR genes [13]. Thus, the ability of *N. caninum* to modulate innate immune responses should be determined in bovine MØs.

Recent *in vitro* studies carried out by our group have shown the capacity of *Neospora* to survive and grow in bovine monocyte-derived MØs by circumventing lysosomal degradation and, for the first time, have shown isolate-dependent differences regarding parasite behaviour in this host cell and the cellular response to infection. The *N. caninum* Nc-Spain7 and Nc-Spain1H isolates, which have shown significant differences *in vitro* and *in vivo* models were used for cell infection. More specifically, Nc-Spain7 shows higher invasion and proliferation rates *in vitro* than Nc-Spain1H [14–16]. In pregnant mice and bovine models, higher transplacental transmission rates and fetal/neonatal mortality were detected after Nc-Spain7 infection than Nc-Spain1H infection [17–21]. *In vitro*, Nc-Spain7 exhibited higher capacities to invade, survive and replicate in bovine MØs than the low-virulence Nc-Spain1H. Moreover, Nc-Spain7 infection induced lower ROS production, *IL10* and *IL12B* expression by MØs than Nc-Spain1H infection, which also resulted in decreased IFN-γ release by activated lymphocytes [14]. These results suggest that *N. caninum* is able to modulate host defences in order to survive and be transmitted in a virulence-dependent manner, and differences in pathogenesis between isolates may be highly related to their ability to induce or subvert protective immune responses in the host.

Transcriptome analysis has been demonstrated to be a suitable approach for studying *N. caninum* host-parasite interactions in target cells [22]. We have previously demonstrated that isolates of different virulence show specific tachyzoite gene expression profiles in bovine macrophages. Specifically, the low-virulence isolate Nc-Spain1H showed enhanced expression of genes encoding for surface antigens, potentially implicated in induction of pro-inflammatory immune responses, and genes related to the bradyzoite stage. On the other hand, the upregulated genes in the high-virulence isolate Nc-Spain7 were related to parasite growth and redox homeostasis [23].

Here, to investigate the molecular basis underlying the innate responses in MØs against *N. caninum* and the effects of parasite manipulation of the host cell environment, we studied the transcriptional profile of bovine monocyte-derived MØs infected with high- (Nc-Spain7) and low- (Nc-Spain1H) virulence *N. caninum* isolates, revealing the main processes involved in MØ activation and modulation.

## Methods

### Bovine monocyte isolation and *in vitro* macrophage differentiation

Monocyte-derived MØs were obtained from peripheral blood from a healthy adult Holstein dairy cow that tested negative for infectious bovine rhinotracheitis virus (IBRV), bovine viral diarrhoea virus (BVDV) and *N. caninum*, following the protocol previously described by García-Sánchez et al. [14]. Briefly, peripheral blood mononuclear cells (PBMCs) were separated by density gradient with Histopaque 1077 (Sigma-Aldrich, St. Louis, MO, USA) and monocytes were isolated using microbeads conjugated to mouse anti-human CD14 antibodies (Miltenyi Biotec Ltd., San Diego, CA, USA). Monocytes were seeded in 6-well culture plates at a density of 3 × 10^6^ cells/well and incubated with 100 ng/ml recombinant bovine GM-CSF (Kingfisher Biotech Inc, St. Paul, MN, USA) at 37 °C and 5% CO2 for 5 days. After 5 days of culture, morphological and functional characteristics compatible with MØs [14] were checked. Cells were harvested, re-seeded at 3 × 10^6^ cells/well in 6-well culture plates, and incubated for 24 h prior to infection.

### Parasite culture and macrophage infection

*Neospora caninum* tachyzoites of both isolates were routinely maintained in an MA-104 cell line culture as described previously [24], keeping in the parasites at a low number of culture passages (< 15) to minimize potential changes in virulence [25]. Tachyzoites used for MØ infection were collected from 3-day-growth cultures, when at least 80% of the parasites were still intracellular, and purified with PD-10 Desalting Columns (GE Healthcare, Chicago, USA) as described previously [16]. Cells were inoculated before 1 h of parasite harvest to minimize loss of viability. A multiplicity of infection (MOI) of three was selected for the inoculation of macrophages with each isolate, to obtain the highest number of infected cells (50–60%) while maintaining cell integrity as described previously [14]. The absence of differences in the percentage of infected cells between groups was confirmed by infecting 3 × 10^5^ cells seeded on coverslips in parallel and under the same conditions and determining the infection rate following the protocol described previously [14]. MØs were also inoculated with the same MOI of 3 with heat-killed (HK) *N. caninum* parasites to study the cell response against *N. caninum* antigens *versus* live tachyzoite infection. HK tachyzoites were obtained by mixing equal quantities of Nc-Spain7 and Nc-Spain1H tachyzoites previously killed by incubation at 56 °C for 30 min. Loss of viability was checked by trypan blue exclusion prior to culture inoculation and by reverse transcription PCR at one-week post-infection as previously described [14]. Non-infected MØs were included as control samples.

At 8 h post-infection (hpi), cells were recovered by cell scraping and centrifuged at 1350×*g* for 10 min at 4 °C. The resulting pellet was resuspended in 300 µl of RNA later (Thermo Fisher Scientific, Madrid, Spain) and stored at − 80 °C until RNA extraction. All analyses were performed with three biological replicates obtained from three independent experiments, each one separated by two weeks. Each replicate was obtained from 3 wells (9 × 10^6^ MØs) inoculated for each condition (Nc-Spain7, Nc-Spain1H, HK and non-infected).

### RNA extraction and RNA-seq

Total RNA was extracted using the Maxwell 16 LEV simply RNA Purification Kit (Promega, Madison, CA, USA). DNase I was added during the extraction process, following the manufacturer’s instructions. RNA integrity was determined by electrophoresis on a 1% agarose gel stained with GelRed (Biotium, Hayward, CA, USA) and visualized under UV light. The quality and quantity of the total RNA obtained was determined in a Bioanalyzer 2100 (Agilent, Redwood City, CA, USA) and a Qubit 2.0 (Thermo Fisher Scientific). An average of 10 μg of RNA were obtained per sample. RNA purity, determined using the ratio of absorbance at 260 nm/280 nm and 260 nm/230 nm, showed values of 2.11 ± 0.03 and 2.18 ± 0.06, respectively. All samples had an RNA integrity number (RIN) between 9.5 and 10.

Twelve samples were sequenced individually by RNA-seq, which consisted of three biological replicates from each of the following samples: MØs inoculated with Nc-Spain7 (MØ7), Nc-Spain1H (MØ1H), HK tachyzoites (MØHK) and non-infected MØs (MØC). The poly(A) + mRNA fraction was isolated from total RNA, and cDNA libraries were obtained following Illumina’s recommendations. Briefly, poly(A) + RNA was isolated on poly-T oligo-attached magnetic beads and chemically fragmented prior to reverse transcription and cDNA generation. The cDNA fragments were then subjected to a repair process, addition of a single ‘A’ base to the 3’ end and ligation of the adapters. Products were purified and enriched by PCR to create the final indexed double stranded cDNA library. The quantity of the libraries was determined by real-time PCR in a LightCycler 480 (Roche, Mannheim, Germany), and their quality was analysed in a Bioanalyzer 2100, High Sensitivity assay. Prior to cluster generation in cBot (Illumina, San Diego, CA, USA), equimolar pooling of the libraries was performed. The cDNA library pool was sequenced by paired-end sequencing (100 × 2) in an Illumina HiSeq 2000 sequencer (Illumina).

### Computational analysis of RNA-seq data

Data quality assessment was performed using the FastQC tool (http://www.bioinformatics.babraham.ac.uk/projects/fastqc). The raw paired-end reads were mapped against the *Bos taurus* genome version UDM3.1 (NCBI: GCA_000003055.3) obtained by the ENSEMBL/NCBI database (http://www.ensembl.org/), using the TopHat2 v2.1.0 algorithm [26]. Low-quality reads were eliminated by the use of Picard Tools (http://picard.sourceforge.net) and selected high-quality reads were assembled and identified through the recommended algorithm in Cufflinks v2.2.1 [27]. The gene quantification process was carried out by the htseq_count 0.6.1p1 tool [28]. The Cufflinks method [27] was used for the quantification and determination of differential expression of the isoforms.

### Differential expression determination, functional enrichment analysis and network analysis

The correlation between samples of the same condition was determined in the statistical software R (http://www.r-project.org) and the whole transcriptome normalized by the size of the library was considered to accept the samples as biological replicates.

The differential expression between sample groups (MØ1H *vs* MØC; MØ7 *vs* MØC; MØHK *vs* MØC; and MØ1H *vs* MØ7) was studied by the recommended DESeq2 algorithm [29], using a binomial negative distribution for the determination of the statistical significance [30]. Genes and isoforms with a fold change (FC) ≥ 1.5 an FDR-adjusted [31] *P*-value (*P*adj) ≤ 0.05 were considered differentially expressed (DE).

For functional enrichment, a hypergeometric test was employed using the human orthologue obtained from the *B. taurus* annotation. Differentially expressed sets from the different study groups were processed using ClusterProfiler [32], a Bioconductor package, to search for biological processes involved. This tool screens for genes in specific databases (i.e. Gene Ontology (GO), Kyoto Encyclopedia of Genes and Genomes (KEGG), Reactome, etc.) to evaluate biological annotations that rise as over-represented. Categories with a *P*adj ≤ 0.05 for each condition were selected. The lists of generated GO terms were summarized in clusters by removing redundant GO terms using the REVIGO tool [33]. The Database for Annotation, Visualization and Integrated Discovery (DAVID) [34] was used to investigate the functional annotation or biological meaning of specific genes of interest. KEGG maps were generated using the R package *Pathview* [35].

### Transcriptomic validation by RT-qPCR

Three additional biological replicates for each condition obtained from three independent experiments were collected and prepared as described for RNA-seq analysis. cDNA was reverse transcribed from extracted RNA using the master mix SuperScript VILO cDNA Synthesis Kit (Invitrogen, Paisley, UK). The expression of selected genes of interest was measured by quantitative real-time PCR (qPCR) using four serial dilutions of each sample (1:20; 1:80; 1:320; and 1:1280) and normalized to the housekeeping gene beta actin (ACTB, ENSBTAG00000026199) and glyceraldehyde-3-phosphate dehydrogenase (GAPDH, ENSBTAG00000014731). Reactions were performed in a 7500 Fast Real-Time PCR System (Applied Biosystems, Foster City, CA, USA) using Power SYBR PCR Master Mix (Applied Biosystems) with the following amplification conditions: 95 °C for 10 min followed by 40 cycles at 95 °C for 15 s and 60 °C for 1 min. Quantitative curves showed a *R*^2^ ≥ 0.992; slope values varied from − 3.67 to − 3.13). Genes used for transcriptomic validation were selected from the most representative signaling pathways enriched from *N. caninum* live infection. Relative gene expression of MØ7, MØ1H and MØHK was calculated by comparing with the expression in MØC using the 2^-ΔΔCq^ method [36]. Primers are listed in Additional file 1: Table S1.

### Immunofluorescence analysis of NF-ƙB p65 nuclear translocation

In a new assay, the induction of NF-ƙB activation by *N. caninum* infection was investigated by analysing the nuclear translocation of the NF-ƙB p65 transcription factor. MØs were seeded on poly-l-lysine-coated coverslips (Sigma-Aldrich) at a density of 3 × 10^5^ cells/coverslip and inoculated with Nc-Spain7, Nc-Spain1H or HK tachyzoites at an MOI of three. Non-infected MØs were used as a negative control. Cultures were fixed at 8 hpi with 0.05% glutaraldehyde and 3% paraformaldehyde, permeabilized with 0.25% Triton X-100 for 20 min at 37 °C, and blocked with 3% bovine serum albumin (BSA; Roche, Mannheim, Germany) for 1 h at room temperature (RT). Cells were labeled with anti-NF-ƙB p65 rabbit polyclonal antibody (Sigma-Aldrich) at 2 µg/ml in 1% BSA for 3 h. Then, Alexa Fluor 488-conjugated goat anti-rabbit IgG (Thermo Fisher Scientific) was used as a secondary antibody at a dilution of 1:400 for 30 min at RT. Parasites were stained using hyperimmune mouse antiserum directed against *N. caninum* tachyzoites (1:200) as the primary antibody [37] for 1 h and Alexa Fluor 594-conjugated goat anti-mouse IgG (Thermo Fisher Scientific) as the secondary antibody for 30 min at RT. The nuclei were stained by washing the cells with DAPI (Thermo Fisher Scientific) at 1:5000 in PBS, and the coverslips were embedded in Fluoroprep (BioMerieux, Marcy-l’Étoile, France). Images were acquired with a Leica TCS SPE confocal microscope with a 40× objective by using the laser lines 405 nm, 488 nm and 547 nm, and overlaid using Photoshop CS6 v13.0 (Adobe Systems Incorporated, San José, CA, USA). Determination of nuclear/cytoplasm mean fluorescence ratios (Fn/c) by image analysis was carried out as described by Sánchez-Aparicio et al. [38], using the Image J1.51n software: Fn/c = Fn–Fb)/(Fc–Fb) where Fn is the nuclear fluorescence, Fc is the cytoplasmic fluorescence, and Fb is the background fluorescence. Cells exhibiting an unusual morphology, size or expression levels were discarded. A total of 100 cells were analyzed by condition by each of three biological replicates. The statistical significance of the data was evaluated by one-way ANOVA, followed by Tukey’s *post-hoc* test for multiple comparisons, using GraphPad Prism 7 v7.04 (GraphPad Inc, San Diego, CA, USA). Differences were considered statistically significant when *P* < 0.05.

## Results

### Sequencing and mapping of RNA-seq data

Nearly 600 million reads were produced, and between 36 and 59 million were obtained from each sample. Between 80–100% of reads were mapped against the reference *B. taurus* genome. Data quality control analysis of duplication studies indicated the lack of degradation of the starting biological material as well as the absence of significant deviations in the sequencing process. In addition, distribution analysis of normalized data showed a correct distribution of biological replicates with no outlier samples. Additional file 2: Table S2 shows the information obtained for each sample in the sequencing process, i.e. the number of total reads, mapped reads and splice reads (related to the capability of the system to detect isoforms and splicing events). Values in the range of 80–100% were observed in the percentage of mapped reads. The gene expression of MØ1H, MØ7, MØHK and MØC is shown in Additional file 3: Table S3.

### Differential expression analysis of *Bos taurus* genes displays different interactions between live and heat-killed *Neospora caninum* with bovine macrophages

A differential expression analysis was performed between *N. caninum*-inoculated MØs (MØ1H, MØ7, MØHK) and MØC (Additional file 4: Tables S4–S7). A total of 3127 differentially expressed genes (DEGs) were identified in the MØ1H-MØC comparison, of which 1538 were upregulated and 1589 were downregulated in MØ1H (Additional file 4: Table S4). A total of 2565 DEGs were found in the MØ7-MØC comparison, with 1292 upregulated and 1273 downregulated in MØ7 (Additional file 4: Table S5). Finally, 1496 DEGs were found in the MØHK-MØC comparison, with 720 upregulated and 776 downregulated in MØHK (Additional file 4: Table S6).

**Fig. 1:**
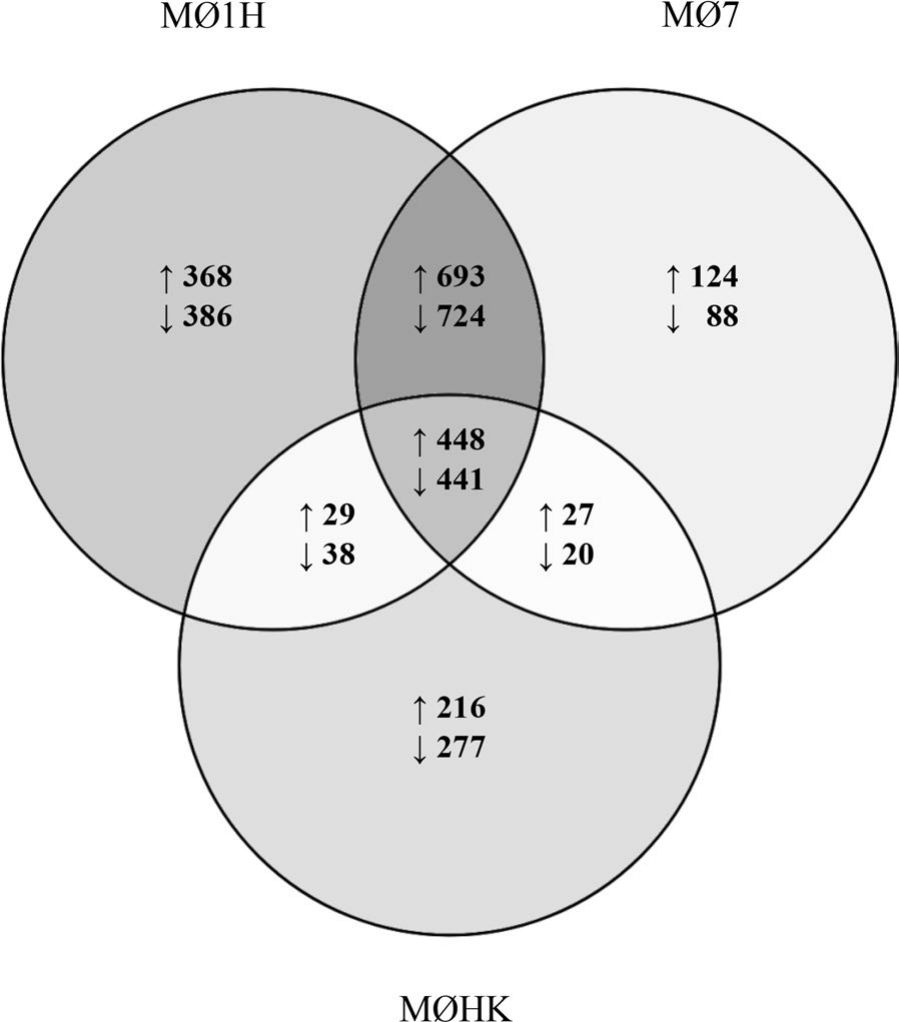
Venn diagram representing DEG between the three groups of *N. caninum* inoculated macrophages *versus* non-infected cells. The diagram shows the number of unique and shared DEG (up- and downregulated) in bovine macrophages infected for 8 h with live tachyzoites of *N. caninum* Nc-Spain7 (MØ7) and Nc-Spain1H (MØ1H), and inoculated with heat-killed tachyzoites (MØHK) in relation to non-infected cells. Genes were considered as differentially expressed when presented a FC ≥ 1.5 and with a *P*adj ≤ 0.05. Results were obtained from three biological replicates for each condition

A Venn diagram was generated to illustrate similarities and differences in the DEGs between the three groups of inoculated MØs and MØC. A common repertoire of genes was regulated under the three conditions, although a bigger number of DEGs was observed with live infection (MØ1H and MØ7), and especially with the MØ1H (Fig. 1).

**Fig. 2:**
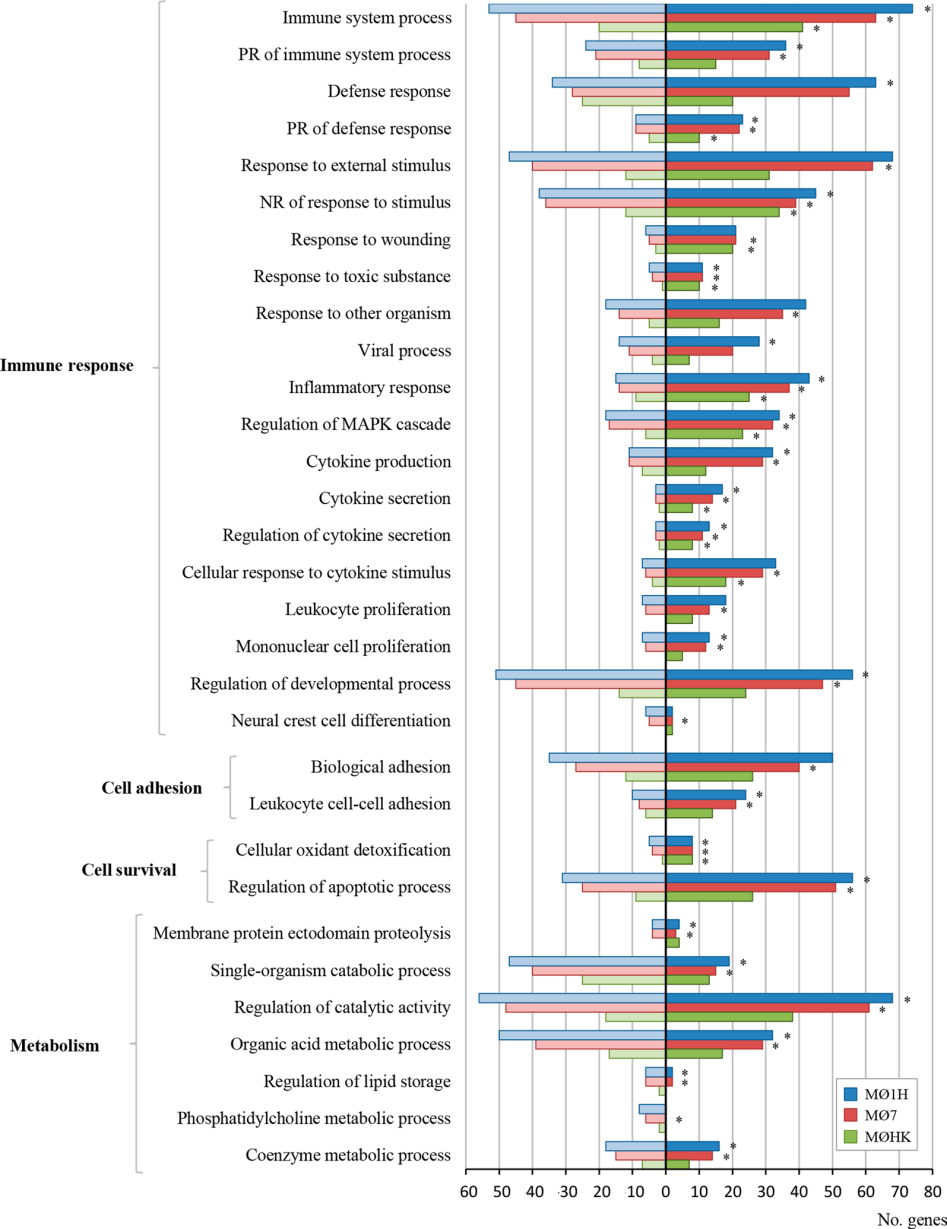
GO terms enriched from DEG in macrophages inoculated with *N. caninum versus* non-infected cells. The graph shows the GO terms enriched from DEG of bovine macrophages infected for 8 h with *N. caninum* Nc-Spain7 (MØ7), Nc-Spain1H (MØ1H), and inoculated with heat-killed tachyzoites (MØHK) in relation to non-infected cells. The x-axis represents the number of DEG mapped for each GO term or pathway. Dark bars indicate upregulated genes and light bars downregulated genes. Asterisks indicate GO terms enriched with a *P*adj ≤ 0.05, considered statistically significant. Results were obtained from three biological replicates for each condition

**Fig. 3:**
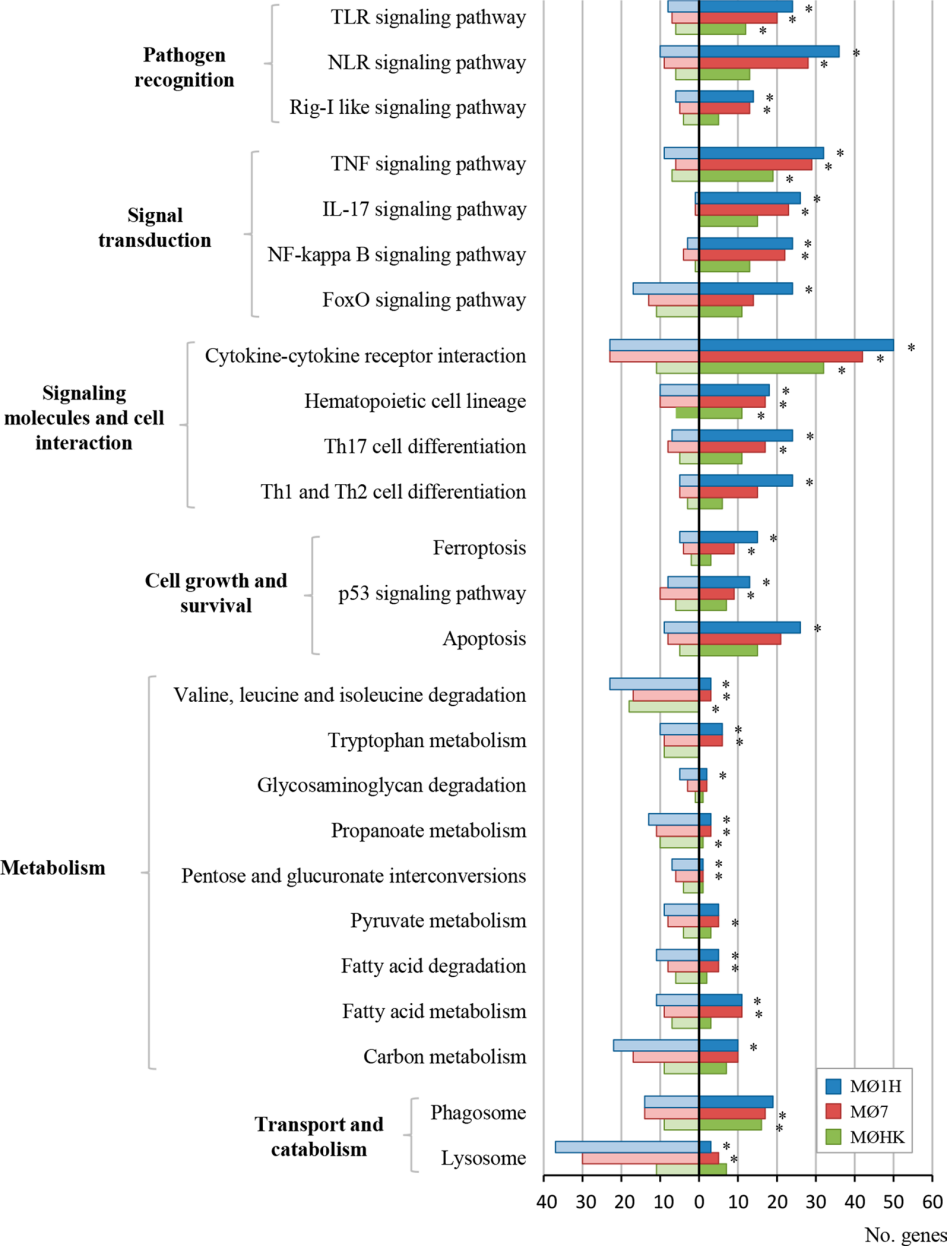
KEGG pathways enriched from DEG in macrophages inoculated with *N. caninum versus* non-infected cells. The graph shows the KEGG pathways [86] enriched from DEG of bovine macrophages infected for 8 h with *N. caninum* Nc-Spain7 (MØ7), Nc-Spain1H (MØ1H), and inoculated with heat-killed tachyzoites (MØHK) in relation to non-infected cells. The x-axis represents the number of DEG mapped for each pathway. Dark bars indicate upregulated genes and light bars downregulated genes. Asterisks indicate pathways enriched with a *P*adj ≤ 0.05, considered statistically significant. Results were obtained from three biological replicates for each condition

**Fig. 4:**
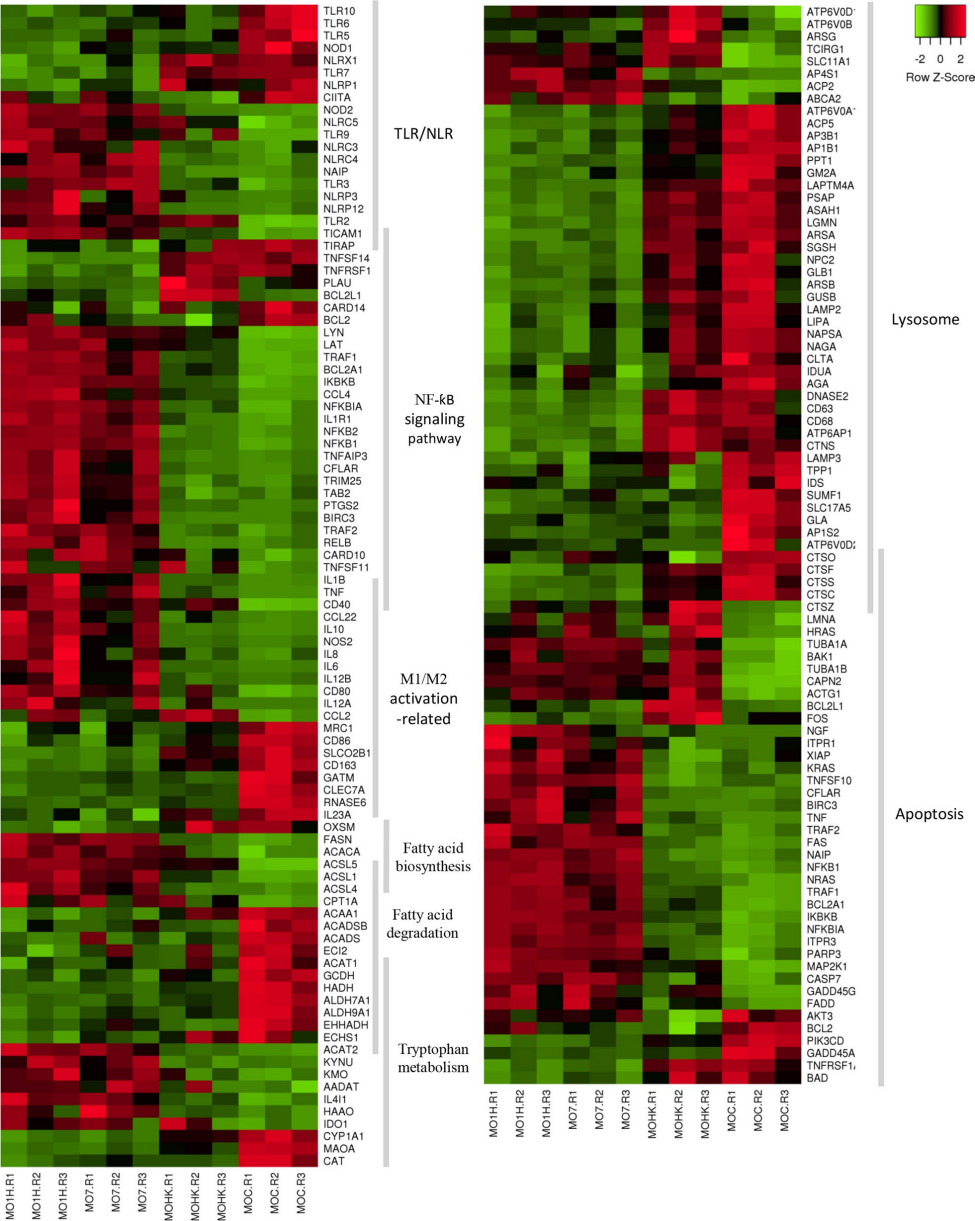
Clustering of DEG in *N. caninum* infected macrophages. Heatmap of a selection of DEG showing row Z-scores based on expression data of three replicates (R1–R3) of MØs challenged for 8 h with Nc-Spain1H (MØ1H), Nc-Spain7 (MØ7), HK tachyzoites (MØHK) and non-infected MØs (MØC). The heatmap was generated using Heatmapper (http://www2.heatmapper.ca). Genes are grouped according to functional categories and clustered into each category by the Pearson computing distance method. mRNA expression values of genes included in the figure are available in Additional file 3: Table S3

To investigate the differential transcriptional response in bovine MØ1H, MØ7 and MØHK, the Gene Ontology (GO) analysis related the Biological Process (BP) domain was carried out for each condition (Additional file 5: Tables S8–S10). Enrichment analysis of the DEGs resulted in 54 GO terms grouped in 23 clusters for the MØ1H-MØC comparison, 52 GO terms in 25 clusters for the MØ7-MØC comparison, and 42 GO terms in 18 clusters for the MØHK*-*MØC comparison (Additional file 6: Table S11). The most representative clusters with GO terms related to the immune response, cell adhesion, cell survival and metabolism are shown in Fig. 2. As expected, a higher representation was observed in inoculations with live tachyzoites than with HK parasites, and interestingly, higher in infection with the low-virulence isolate Nc-Spain1H than with the Nc-Spain7 isolate. MØ1H-MØC, MØ7-MØC and MØHK-MØC comparisons were also mapped on Kyoto Encyclopedia of Genes and Genomes (KEGG) pathways (Additional file 7: Tables S12-S14). KEGG pathway analysis of the DEGs revealed 36 pathways for MØ1H-MØC (Additional file 7: Table S12), 35 for MØ7-MØC (Additional file 7: Table S13) and 12 for MØHK-MØC (Additional file 7: Table S14). Important pathways enriched exclusively in live infections (MØ7 and MØ1H) were those related to pathogen recognition (NLR and RIG-I-like signalling pathways), signal transduction and cell interaction (IL17 signalling pathway, NF-ƙB signalling pathway and Th17 cell differentiation). In addition, those involved in cell growth and survival (ferroptosis and the p53 signalling pathway), metabolism (tryptophan metabolism, pentose and glucuronate interconversions, and fatty acid metabolism) and transport and catabolism involving “lysosome” were also modulated only by live parasites. Among these, remarkably metabolism and lysosome-related pathways appeared to be downregulated (Fig. 3). The unique DEGs for MØ7 and MØ1H were also studied, but no significant results were obtained in any of the enrichment analyses. Figure 4 shows differences in the expression of a selection of genes grouped into functional categories of interest.

Quantitative real-time PCR was used to validate the RNA-Seq results. The expression of 15 selected genes showed similar profiles for both RNA-seq and real-time PCR techniques and similar FCs for the two housekeeping genes used for validation (Additional file 8: Figure S1).

### Pattern recognition receptor expression is differentially regulated by live *Neospora caninum*

A key element in the initiation of the innate immune response is the recognition of the pathogen by APCs by means of PRRs. Toll-like receptors (TLRs) *TLR2* and *TLR9* were upregulated, whereas *TLR5*, *TLR6* and *TLR10* were downregulated in MØ7, MØ1H and MØHK. Interestingly, there was upregulation of *TLR3* and inhibition of *TLR7* expression exclusively in those MØs infected with live parasites (MØ7 and MØ1H). In addition, nod-like receptors (NLRs) expression was modulated; *NOD1* was downregulated and*NLRC5* was upregulated in MØ7, MØ1H and MØHK whereas *NAIP*, *NOD2*, *NLRC3*, *NLRC4* and *NLRP12* were upregulated, and *NLRX1* was downregulated in MØs infected with live tachyzoites of both isolates. Finally, *NLRP1* was downregulated and *NLRP3* was upregulated only in MØ1H (Fig. 4).

### Activation of the NF-ƙB signalling pathway is induced in bovine macrophages during *Neospora caninum* infection

**Fig. 5:**
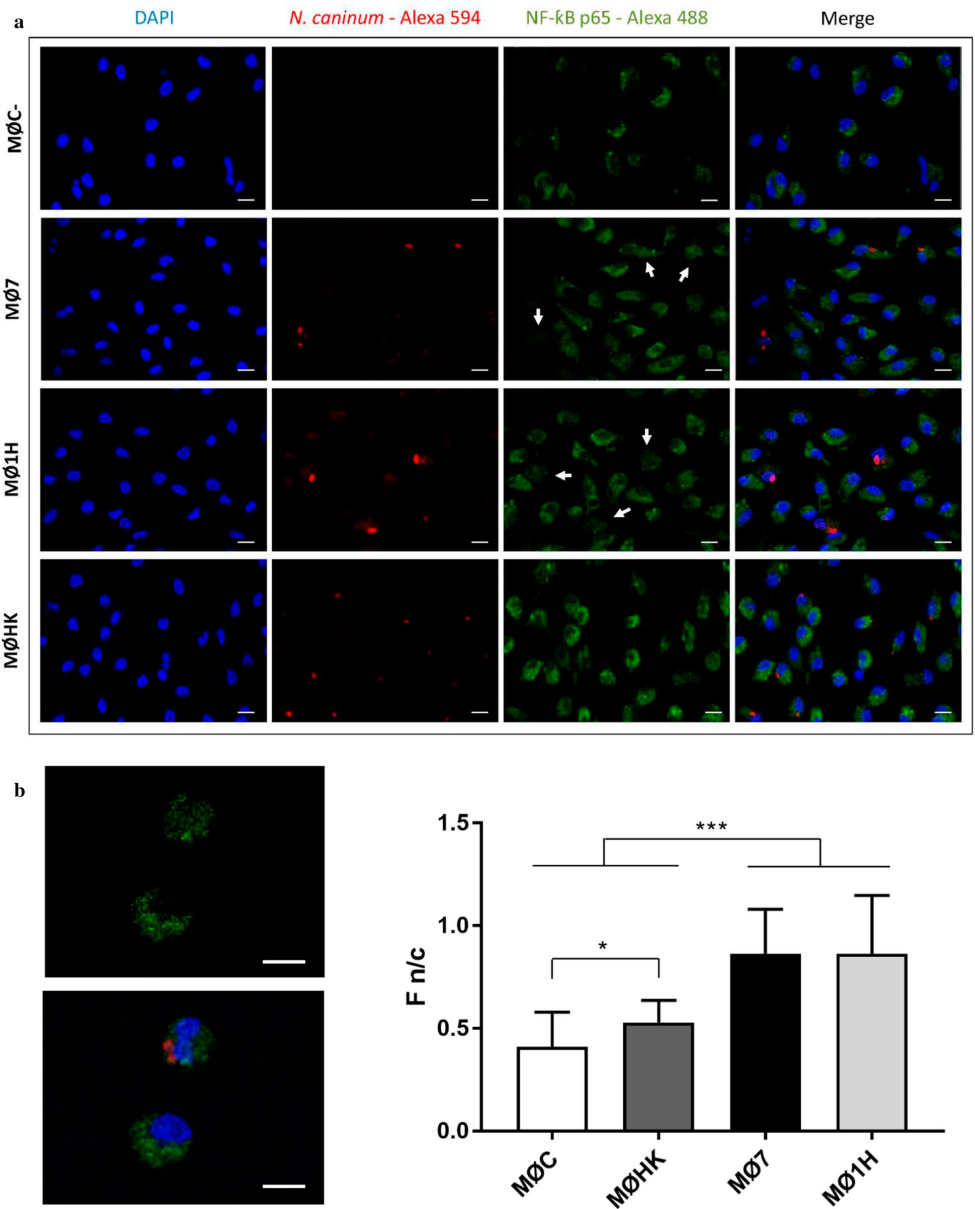
Activation of NF-ƙB upon *N. caninum* active invasion. **a** Representative micrographs of non-infected MØs (MØC) and MØs challenged for 8 h with Nc-Spain7 (MØ7), Nc-Spain1H (MØ1H) or HK tachyzoites (MØHK). NF-ƙB p65 is stained in green (Alexa Fluor 488), parasites in red (Alexa Fluor 594) and nuclei in blue (DAPI). Arrows exemplify *N. caninum* infected cells, where co-location of NF-ƙB p65 and nuclei is observed. **b** Image magnifications where nuclear translocation of NF-ƙB p65 from cytoplasm to nucleus is shown exclusively in the *N. caninum* infected-MØ. The upper image shows exclusively the NF-ƙB p65 staining. The image below is a merged image of NF-ƙB p65, nuclei and parasites staining. **c** The bar graph indicates the nuclear/cytoplasmic mean fluorescence intensity ratios (F n/c) ratio of MØC, MØ7, MØ1H and MØHK expressed as the mean ± SD. Asterisks indicate significant differences (*P < 0.024, ***P < 0.0001). Results were obtained from three biological replicates for each condition. *Scale-bars*: **a**, **b**, 10 µm

**Fig. 6:**
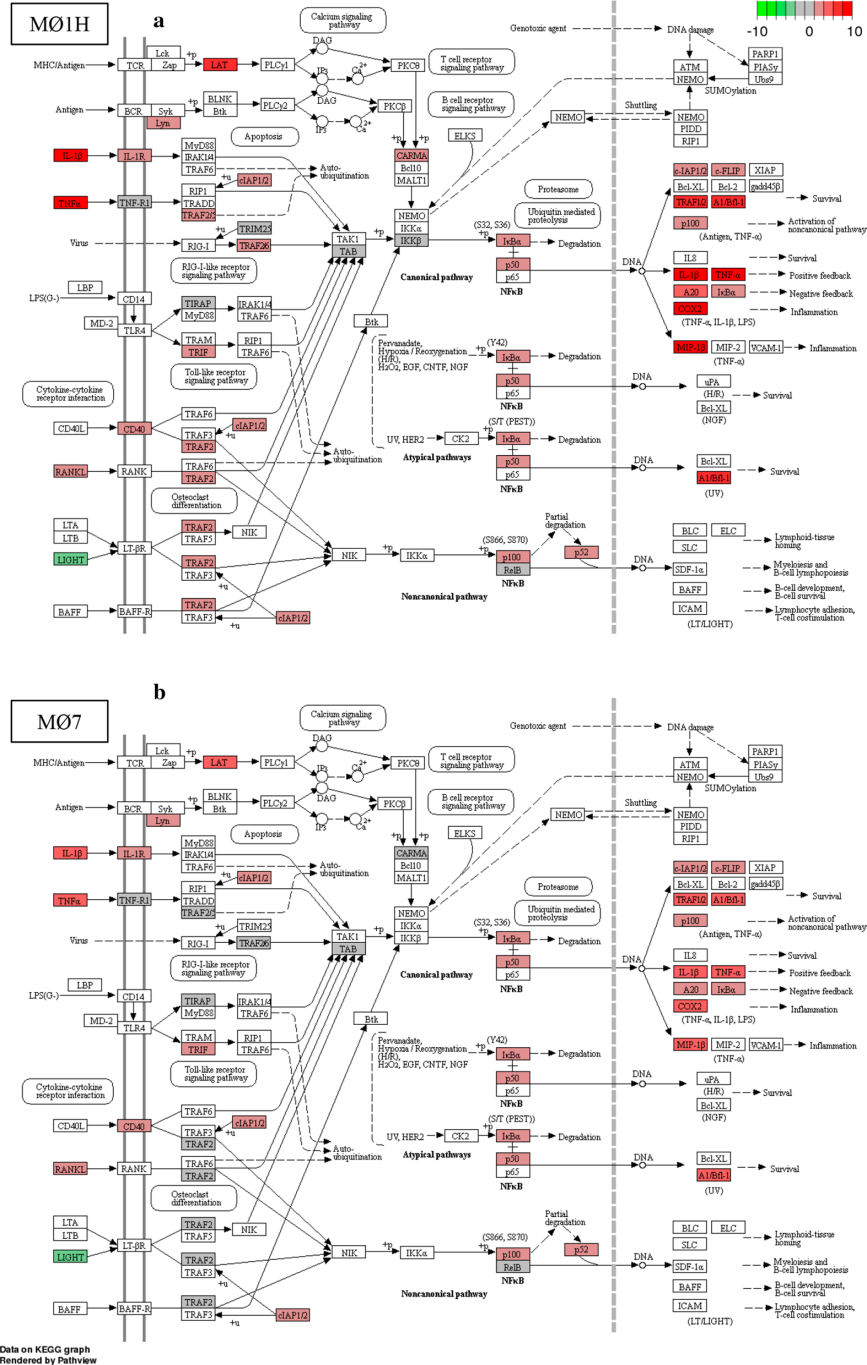
The KEGG pathway maps [86] represent the NF-ƙB signalling pathway in which DEG in (**a**) bovine macrophages infected with Nc-Spain1H and (**b**) Nc-Spain7 *versus* non-infected macrophages are highlighted. Upregulated genes are represented in red and downregulated genes are represented in green. The color intensity corresponds to the level of up- or downregulation. Results were obtained from three biological replicates for each condition

Pathogen recognition by TLRs and NLRs leads to the activation of signalling cascades that stimulate host defences [13, 39]. The NF-ƙB signalling pathway was observed to be significantly upregulated exclusively in MØs infected with live tachyzoites (MØ1H and MØ7) (Fig. 3). NF-ƙB activation by *N. caninum* infection was also functionally demonstrated in this study by examining the translocation of NF-ƙB p65 from the cytoplasm to the nucleus in MØ1H and MØ7 but not in MØC (Fig. 5a, b). Nuclear/cytoplasm mean fluorescence ratios (Fn/c) (Fig. 5c) were higher for the three groups of *N. caninum*-inoculated macrophages (MØ1H, MØ7 and MØHK) than for MØC (ANOVA, *F*_(3, 219)_ = 69.87, *P* < 0.0001–0.024). However, NF-ƙB p65 translocation was highly induced by live infection (MØ1H and MØ7) *vs* MØHK (ANOVA, *F*_(3, 219)_ = 69.87, *P* < 0.0001). Although a very similar response was observed regarding the DEGs implicated in this pathway for MØ1H and MØ7, for nearly all of them, the levels of mRNA regulation were higher for MØ1H, and expression was higher for the adaptor *TRAF2*, which results in an enhanced expression of pro-inflammatory cytokines *TNF*, *IL1B*, *IL6* and *IL12B* as can be observed in Fig. 6a, b.

MAPK pathways ERK, JNK and p38 in MØs, which have been previously related to murine resistance against *N. caninum* [7, 40], were also investigated. In our study, the ERK and JNK pathways were not upregulated in MØs inoculated with either live or HK tachyzoites. Although the p38 pathway was not enriched in the KEGG analysis, the p38 genes *MAPK11* and *MAPK12* (encoding p38-β and p38-γ MAPK, respectively) appeared upregulated in MØ7, MØ1H and MØHK. In addition, *MAPK14* (encoding p38-α MAPK) mRNA levels were not altered in MØ7 and MØHK, whereas they were downregulated in MØ1H.

### *Neospora caninum* infection induces macrophage polarization towards the M1 phenotype

MØs exhibit a high degree of plasticity and are able to respond to stimuli and polarize to an M1 (classical activation) phenotype or an M2 (alternative activation) phenotype associated to predominant Th1 and Th2 responses, respectively [41]. *Neospora caninum*-inoculated MØs (MØ7, MØ1H and MØHK) showed a predominantly M1 phenotype, characterized by an enhanced expression of pro-inflammatory cytokines *TNF*, *IL6*, *IL1B*, and other M1 markers (*CCL2*, *CD80* and *CD40*) and downregulation of the M2 markers *CLEC2A*, *MRC1*, *CD163*, *RNASE6*, *GATM* and *SLCO2B1.* However, the M2-associated chemokine *CCL22* were also upregulated.

Live tachyzoites infection (MØ7 and MØ1H) also induced upregulation of *NOS2* and pro-inflammatory *IL12B* (that encodes IL12p40) (Fig. 4, Additional file 4: Tables S4, S5).

### *Neospora caninum* circumvents phagolysosome activity and modulates macrophage apoptosis

The expression profiles indicated lysosome pathway inhibition and macrophage apoptosis modulation in MØ7 and MØ1H but not MØHK. “Apoptosis” and “Regulation of apoptotic process” were KEGG pathways and GO terms associated with *N. caninum* infection in this study (Figs. 2, 3), although the first appeared as statistically significant only for Nc-Spain1H infection. Genes annotated as anti-apoptotic and as pro-apoptotic were both upregulated and downregulated (Fig. 4, Additional file 5: Tables S8, S9).

**Fig. 7:**
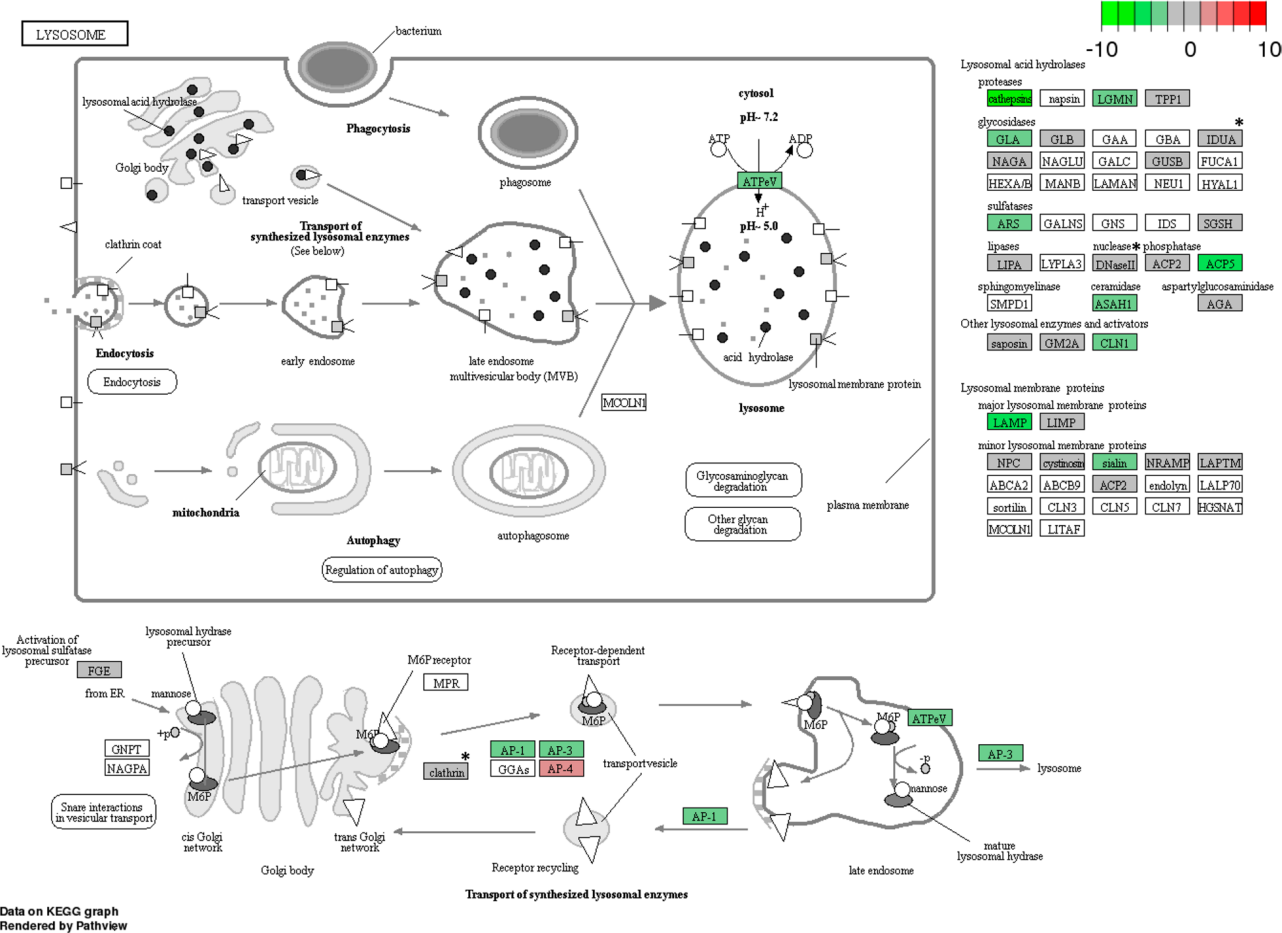
Lysosome pathway in macrophages infected with Nc-Spain1H *versus* non-infected cells. The KEGG pathway map [86] represents the molecular network “Lysosome” in which DEG in bovine macrophages infected with Nc-Spain1H *versus* non-infected macrophages are highlighted. Upregulated genes are represented in red and downregulated genes are represented in green. The color intensity corresponds to the level of up- or downregulation. Results were obtained from three biological replicates for each condition. Asterisks indicate genes differentially expressed upon Nc-Spain1H but not Nc-Spain7 infection

Regarding the lysosome pathway, MØ7 and MØ1H showed the downregulation of genes encoding proteins involved in phagolysosome formation such as V-ATPase, lysosomal-associated membrane proteins, proteins involved in the transport and activation of lysosomal enzymes, and enzymes and peptides with microbicidal effects [42, 43] (Fig. 7, Additional file 7: Tables S12, S13).

### *Neospora caninum* infection markedly impacts host metabolic pathways

**Fig. 8:**
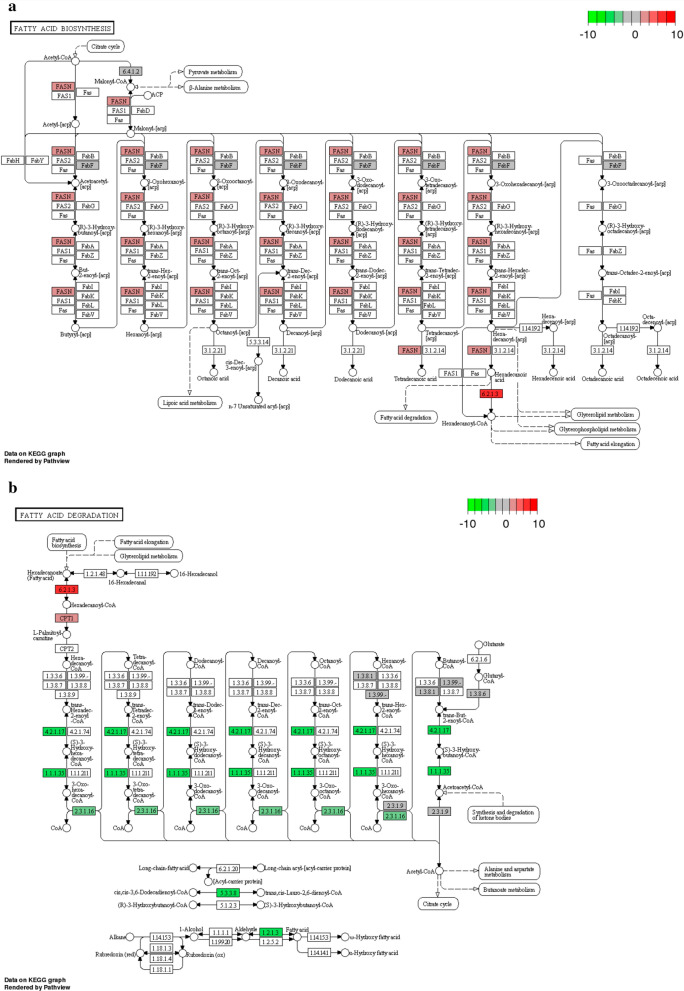
Fatty acid metabolic pathways in macrophages infected with Nc-Spain1H *versus* non-infected cells. The KEGG pathway maps [86] represent the molecular networks Fatty acid biosynthesis (**a**) and Fatty acid degradation (**b**) in which DEG in bovine macrophages infected with Nc-Spain1H *versus* non-infected macrophages are highlighted. Upregulated genes are represented in red and downregulated genes are represented in green. The color intensity corresponds to the level of up- or downregulation. Results were obtained from three biological replicates for each condition. Similar regulation was observed for Nc-Spain7 infection

Enrichment analysis showed an impact of *N. caninum* on the host cell metabolism. Of particular interest, live parasites (MØ7 and MØ1H) infection regulated the KEGG pathways “Fatty acid metabolism”, “Fatty acid degradation”, “Pentose and gluconorate interconversions” and “Tryptophan metabolism”; and amongst GO terms “Regulation of catalytic activity”, “Organic acid metabolic process” and “Regulation of lipid storage” (Figs. 2, 3). Because fatty acid synthesis that occur in cytoplasm and fatty acid degradation in mitochondria have been previously related with MØ polarization [44], we further studied the impact of *N. caninum* infection in these cellular processes. In the present study we observed a clear downregulation of fatty acid degradation together with upregulation of fatty acid synthesis (Figs. 4, 8).

In addition, “Tryptophan metabolism” was also modulated in *N. caninum* infected MØs (Fig. 3, Additional file 7: Tables S12, S13). The majority of tryptophan is metabolized by the Kyneurine pathway by two different enzymes, indoleamine-2,3-dioxygenase 1 (*IDO1*) and tryptophan-2,3-dioxygenase (*TDO*) giving place to different catabolites collectively called kynurenines that can act as immunoregulatory factors [45]. Enzymes involved in Tryptophan degradation were upregulated in *N. caninum* infected MØs (Additional file 7: Tables S12, S13). Remarkably, *IDO1* was upregulated only in MØ1H (Additional file 4: Table S4).

### Differences between *Neospora caninum* isolates define a mechanism for evasion of the immune response of the highly virulent isolate Nc-Spain7

A differential expression analysis between MØ1H *vs* MØ7 resulted in four DEGs: C-C motif chemokine 4 (CCL4), C-X-C motif chemokine 6 (CXCL6) and metallothionein-2 (MT2) were upregulated in MØ1H; and coenzyme Q8A (ADCK3) was upregulated in MØ7 (Additional file 4: Table S7). However, a higher number of differentially regulated genes were found for the Nc-Spain1H isolate in the comparison of MØ1H-MØC *versus* MØ7-MØC (Fig. 1). Among the DEGs unique for MØ1H that belong to common pathways shared by MØ1H and MØ7, the following can be highlighted: genes associated with the “NLR signalling pathway” (NLRP1 and NLRP3); “Chemokine signalling pathway” (e.g. BCAR1, GNAI3, GNG4, JAK2, ROCK2, CXCL5, IKBKB, MAP2K1, NRAS and PIK3CG); and “Cytokine-cytokine receptor interaction” (e.g. IL8, TNFRSF8, IFNAR2, IL12A, IL17RA, TNFSF15 and TNFSF8) (Additional file 7: Tables S12, S13). In addition, the pathways “FoxO signalling pathway”, “Th1 and Th2 cell differentiation” “Glycosaminoglycan (GAG) degradation” and “Apoptosis” appeared to be statistically significant only for MØ1H (Fig. 3, Additional file 7: Table S12).

## Discussion

Studies in murine and human MØs have identified signalling pathways implicated in host resistance against *N. caninum* and mechanisms used by the parasite to evade the immune responses mounted by these cells [5–12]. Here, the transcriptional analysis of bovine monocyte-derived MØs infected with high-virulence (Nc-Spain7) and low-virulence (Nc-Spain1H) *N. caninum* isolates has been used as an approach to study if these mechanisms may be also implicated in *N. caninum* interaction with his natural host.

The expression of TLR and NLR, as essential elements implicated in pathogen recognition by the MØs, were first studied. Our results suggest a role of TLR2, TLR3 and TLR9 in *N. caninum* infection in the bovine MØ response. The importance of TLR2 and TLR3 in *N. caninum* recognition has been previously described in murine MØs, whereby they trigger the production of pro-inflammatory cytokines [6, 46, 47]. TLR2 expression is also regulated by *N. caninum* infection in bovine trophoblast cells [22], and immunization with *N. caninum* inactivated antigens induces TLR3 and TLR9 in the maternal-fetal interface of infected pregnant heifers [48].

Conversely, thus far the role of NLRs in neosporosis is unclear, and only two members of these cytoplasmic receptors, NOD2 and NLRP3, have been related to murine MØ responsiveness in previous studies [8, 49]. NOD2 activation in mice has been related to an exacerbation of the inflammatory response, contributing to initial parasite control, but causing tissue damage during acute neosporosis [49]. In the present study, NLRs related to inflammasome formation [39] *NAIP*, *NOD2*, *NLRC4* and *NLRP12* were only over-expressed in cells infected with live tachyzoites of both isolates, suggesting different recognition and thus subsequent responses induced by live and HK tachyzoites, likely because NLRs act as intracellular surveillance molecules. The involvement of all these receptors in *T. gondii* sensing has been reported previously [50–52], and here the results suggest for the first time that they may be involved in the bovine MØ inflammatory response against neosporosis.

Moreover, the major histocompatibility complex (MHC) class II transactivator *CIITA* was over-expressed in MØHK, likely because of the exclusive antigen presentation *via* phagocytosis of inactivated parasites [14], whereas a reduction of MHC II was previously detected in bovine MØs infected with live *N. caninum* [14], which suggests a downregulation of its expression by live parasites. Inhibition of *CIITA* expression may be a mechanism used by *N. caninum* to limit MHC II expression in APCs to evade CD4^+^-mediated immune responses, as previously described for *T. gondii* [53].

Besides, autophagy has been associated with the control of infections by directing intracellular or phagocytosed pathogens to lysosomes for degradation [54]. *NLRX1* and *NOD1* are related to autophagy, the first one located in the mitochondria and involved in ROS production [55], and were downregulated in MØ7 and MØ1H but not in MØHK. Thus, *N. caninum* may inhibit their expression in order to survive in the cell. In fact, a reduction in ROS response has been previously demonstrated for early *N. caninum* infection [14].

NF-ƙB was the main signalling pathway activated in bovine MØs infected by *N. caninum* high and low virulence isolates. Enhanced expression of NLRs related not only to NF-ƙB signalling pathway activation (*NOD2*), but also to NF-ƙB negative regulation (*NLRC3* and *NLRP12*) [39, 56, 57], was observed in MØs infected with live tachyzoites, which points towards a modulation of the inflammatory response by *N. caninum* with the aim of avoiding pathologic consequences due to a persistent activation of NF-ƙB [58].

Pro-inflammatory cytokine production induced by the activation of MAPK pathways ERK, JNK and p38 in MØs has been related to murine resistance against *N. caninum* [7, 40]. In our study, the ERK and JNK pathways were not upregulated in MØs inoculated with either live or HK tachyzoites, suggesting that they are probably not involved in the bovine host immune response against *N. caninum.* Regarding the p38 pathway, Mota et al. [10] proposed p38 MAPK activation as a mechanism used by *N. caninum* to evade murine innate immunity, *via* a pronounced decrease in IL-12p40 production. Curiously, in our study *MAPK14* (encoding p38-α MAPK) mRNA levels were downregulated only in MØ1H, which also showed higher levels of *IL12B* (IL-12p40) expression.

Altogether, our results indicate that NF-ƙB is the main signalling pathway implicated in the pro-inflammatory immune response against *N. caninum* in bovine MØs. In *T. gondii*, differences in the activation of this pathway have been related to parasite type since type II strains induce a higher NF-κB activation than type I and III strains, which affects cytokine expression and virulence [59]. Activation of the NF-ƙB signalling pathway upon *N. caninum* infection has been previously reported in murine MØs, and it has been related to the expression of *N. caninum* 14-3-3 protein [40] and dense granule proteins NcGRA6, NcGRA7 and NcGRA14 [60]. However, the higher levels of mRNA expression found for the DEG of the NF- ƙB signalling pathway in Nc-Spain1H infection seems not to correlate with a higher expression of those dense granule proteins for this isolate [23]. Thus, new studies are necessary to establish the parasite effector molecules involved in NF-ƙB activation in bovine MØs.

It is also known that virulence and evasion mechanisms of the pathogens may affect MØ polarization, with consequences for parasite burden and inflammation-related pathologies [61, 62]. Diverse *T. gondii* strains can induce a different phenotype in MØs, where high virulence has been associated with M2 induction [62]. Here, both high- and low-virulence *N. caninum* isolates induced a predominantly M1 phenotype associated to a pro-inflamatory response required to reduce *N. caninum* proliferation within the host [63]. However, this type of response is believed to be one of the main factors that trigger stage conversion of tachyzoites to bradyzoites. Thus, the pro-inflammatory response would not only reduce parasite loads in the host, but it may also favor parasite encystation for long lasting survival and transmission to definitive host [64]. In support to this, a higher expression of genes related with the bradyzoite stage were found for the low virulence isolate Nc-Spain1H when infecting bovine macrophages [23], which induces a higher pro-inflammatory response by this cell, as has been demonstrated in the present and previous studies [14].

Macrophage polarization is also influenced by metabolism. Manipulation of the host metabolism is achieved to obtain the energy and nutrients that the parasites need to survive and proliferate [65]. Besides, metabolic reactions and processes may control immunological effector functions such as cytokine production in response to pathogens [66]. Inflammatory signals including LPS and IFN-γ, required to generate M1 MØs, have been shown to drive fatty acid synthesis, whereas the inhibition of inflammatory signals required for the differentiation of M2 MØs involves fatty acid degradation [44]. In the present study, we observed a clear downregulation of fatty acid degradation together with upregulation of fatty acid synthesis that may again reflect M1 polarization of *N. caninum*-infected MØs. In addition, “Tryptophan metabolism” was also modulated. Tryptophan metabolism leads to the production of catabolites that can act as immunoregulatory factors [45]. Several enzymes involved in tryptophan degradation were upregulated in MØs infected with both isolates except *IDO1*, which was upregulated only in MØ1H. Because restriction of *N. caninum* proliferation induced by *IDO1* activity has been demonstrated *in vitro* [67], this antiparasitic mechanism of the host cell may be related to the lower proliferation of Nc-Spain1H in bovine MØs [14].

It was demonstrated that both Nc-Spain7 and Nc-Spain1H were able to survive in bovine MØs *in vitro* [14]. The results in the present study point towards modulation of apoptosis and impairing phagolysosome maturation as mechanism used by *N. caninum* to survive and proliferate into the phagocytic cells. Both isolates induced the downregulation of genes related to the maturation of the phagosome to a phagolysosome containing a robust antimicrobial environment [42]. These results support the phenotype previously described for *N. caninum*-infected bovine MØs, where the absence of lysosome activity was demonstrated in parasitophorous vacuoles and was limited to tachyzoites internalized by phagocytosis [14]. Apoptosis, is one of the main defence mechanisms that hosts possess against intracellular pathogens such as *T. gondii* [68, 69]. On the one hand, stress signals provided by the parasite during infection may induce a pro-apoptotic response by the host cell as an innate defense mechanism. On the other hand, obligate intracellular parasites need to hijack apoptosis-regulating cascades in order to suppress or delay cell death to favour parasite replication [69]. Our results indicate that Nc-Spain7 and Nc-Spain1H counteract stress-induced cell death by promoting the expression of certain anti-apoptotic BCL-2 genes, inhibitors of apoptosis (IAPs) and *FLIP* (also named *CFLAR*) among others. This would lead to a decreased release of cytochrome *c* from mitochondria into the cytosol and to a direct interference with caspase processing and function, as has been described for *T. gondii* and other protozoan parasites [68, 69]. In cells infected with *T. gondii*, upregulation of pro-survival genes has been related to NF-κB activation. However, it has been described that *N. caninum* inhibits murine cells apoptosis in the absence of NF-ƙB p65 translocation to the nucleus [70]. The implication of this transcription factor in the observed modulation of apoptosis induced by *N. caninum* isolates in bovine macrophages requires further investigation. Despite both isolates are able to survive intracellularly, differences between isolates were found in their survival rates *in vitro* and in parasite burdens *in vivo* in previous studies [14]. The variations shown in the response of MØs to infection by Nc-Spain7 and Nc-Spain1H may help to understand their differences in pathogenesis.

In the MØ1H *vs* MØ7 comparison, CCL4, CXCL6 and MT2 were upregulated in MØ1H. *CXCL6* and *CCL4* are chemokines for neutrophils and monocytes, and natural killer cells and regulatory T cells, respectively. In addition to their pro-inflammatory effects, CCL4 can also promote homeostasis by interacting with CCR5 receptors [71, 72]. Activation and migration of immune cells *via* CCR5 seems to be essential for controlling *N. caninum* during the acute phase of the disease [73]. The *MT2* gene encodes metallothionein, whose upregulation during infection may serve as a strategy to prevent host tissue damage because of its capability to neutralize ROS [74]. The observed upregulation of *MT2* may be a response to the higher intracellular ROS levels induced in MØs infected by Nc-Spain1H as previously described [14]. *MT2* may also be involved in the regulation of immune response signalling and inflammation by modulating the activation of NF-ƙB, pathogen clearance in MØs and inhibition of apoptosis through mediation of p53 activity [74].

Additionally, a higher impact in host-cell regulation was observed for MØ1H than MØ7. Nc-Spain1H infection but not Nc-Spain7 infection resulted in the enhanced expression by bovine MØs of genes associated with the “NLR signalling pathway”, “Chemokine signalling pathway” and “Cytokine-cytokine receptor interaction”. A stronger cellular stimulation induced by Nc-Spain1H has been previously described *in vitro* in bovine MØs and trophoblast cells, which could be related to a higher abundance of highly immunogenic cell surface proteins in this isolate, activating a more efficient immune response that leads to the control of Nc-Spain1H infection [14, 23, 75]. The NLR NLRP3 expression was upregulated and NLRP1 was downregulated only in the MØ1H, isolate which shows lower parasite survival in bovine MØs infected *in vitro* [14]. Activation of both inflammasome sensors has been related to control of parasite proliferation in murine macrophages and host resistance to *T. gondii* infection [76]. Our results suggest a similar role for NLRP3, which has been previously implicated in limiting parasite growth *via* the Th1 response and IFN-γ induction in *N. caninum* infected mice [8]. However, the role of NLRP1 in sensing *N. caninum* which seems not to correlate with the observed for *T. gondii*, remains to be elucidated. Additionally, the higher levels of mRNA expression found for the DEG of the NF- kB signalling pathway in Nc-Spain1H infection may be related to the observed variations in the expression of pro-inflammatory cytokines such as *TNF*, *IL1B*, *IL6* and *IL12B*. Nc-Spain1H also induced enhanced expression of pro-inflammatory *IL8* and of *IL12A* that was not observed for Nc-Spain7. *IL12A* (IL12p35) and *IL12B* (IL12p40) need to be coordinately expressed to produce active IL-12 (IL12p70) [77]; therefore, reduced expression of *IL12A* would limit IL-12 production in MØ7. This cytokine induces IFN-γ release by natural killer (NK) cells and CD4^+^ T lymphocytes, and both IL-12 and IFN-γ are essential for restricting *N. caninum* intracellular growth [63, 77]. A reduced secretion of IFN-γ was also detected in lymphocytes stimulated with MØs infected with Nc-Spain7 [14]. In addition, *IL23*, a pro-inflammatory cytokine important for controlling *T. gondii* infection [78], was downregulated in MØ7. Altogether, our results may explain the lower induction of IFN-γ release by lymphocytes by MØ7 and the higher ability of Nc-Spain7 to survive in MØs observed *in vitro* [14] and may be related to the higher parasite burdens and the induction of placental lesions and abortions found *in vivo* [20].

In addition, the pathways “Th1 and Th2 cell differentiation”, “GAG degradation”, “FoxO signalling pathway”, and “Apoptosis” appeared to be statistically significant only for Nc-Spain1H infection. Although it is widely accepted that a protective immunity against neosporosis requires a mixed Th1/Th2 response [63], the adequate balance between both has not been defined yet. Thus, this study may help to shed light on the factors implicated in neosporosis pathogenesis. Among the DEG exclusively for MØ1H related to the Th1/Th2 differentiation pathway, beyond *IL12A* and *MAPK14*, it is worth highlighting *GATA3*, that plays an important role in MØ polarization towards an M2 phenotype [79] and *DLL4*, involved in the induction of Th1 differentiation [80]. MØ1H showed downregulation of genes involved in the degradation of chondroitin sulfate (CS) and heparan sulfate (HS), the major Glycosaminoglycan (GAG) expressed on the cell surface membrane [81]. CS and HS mediate the initial interaction of several pathogens including *T. gondii* and *N. caninum* with the host cell [82]. Inflammatory and immuno-regulatory mediators including many chemokines, cytokines, and growth factors interact with these cell-surface GAGs [81], and the ability of pathogens to subvert GAG functions is considered an important virulence mechanism [83]. On this basis, inhibition of HS and CS degradation in MØ1H may be related with a higher abundance of these GAGs in the cell surface, which may partially explain the different induction of pro-inflammatory responses between both isolates. The Forkhead box O (FOXO) signalling pathway regulates the expression of genes involved in apoptosis, cell-cycle control, metabolism and oxidative stress resistance [84]. The Nc-Spain1H isolate, despite showing a lower proliferation rate, induces a higher expression of Th1 cytokines and ROS production [14], which may be associated with a higher activation of stress signals in the cell and therefore expression of genes related to FOXO signalling pathway and apoptosis.

## Conclusions

The study of gene expression profiles in this study has revealed mechanisms implicated in the recognition of *N. caninum* by bovine MØs and in the subsequent immune response. NF-ƙB seems to be an essential signalling pathway implicated in the response against this pathogen of its natural host. Gene expression profile after MØs activation by *N. caninum* showed a metabolic and immune M1 inflammatory phenotype needed for the control of infection. Apoptosis and degradation by lysosomes are processes repressed by *N. caninum* infection, which may guarantee its survival in this cell type. Gene expression modulation by *N. caninum* infection resemble with phenotypic traits previously studied [14]. The present study shows that *N. caninum* is able to modulate MØ host signalling pathways to escape cellular defences and opens up a new avenue for further studies on parasite virulence. The sum of the variations found between the high- and the low- virulence isolates in the expression of genes involved in pathogen sensing, microbial killing, cell survival, chemotaxis or cytokine release may result in important differences regarding the immune responses generated against these two isolates. An expected enhanced but highly regulated early protective response to infection induced by Nc-Spain1H would explain why Nc-Spain1H shows limited infection of placental tissues with no abortion in a bovine model. In contrast, mechanisms of evasion by Nc-Spain7 support the efficient transmission of this isolate to the foetus causing abortion [20]. Further studies are necessary to determine whether *N. caninum* manipulates bovine macrophages at a post-trascriptional level, as has been described for *T. gondii* and other apicomplexan parasites [85], as well as the parasite factors implicated in virulence-related cell modulation.

## Supplementary information

**Additional file 1: Table S1.** Sequences of primers used for transcriptomic validation by RT-qPCR.

**Additional file 2: Table S2.** Mapped and paired reads by sample against the *Bos taurus* genome.

**Additional file 3: Table S3.** RNA-seq results for *Bos taurus* genes in bovine macrophages inoculated with *Neospora caninum* Nc-Spain1H (MØ1H), Nc-Spain7 (MØ7), heat-killed tachyzoites (MØHK), and in non-infected macrophages (MØC).

**Additional file 4: Table S4.***Bos taurus* differentially expressed genes in bovine macrophages infected with *Neospora caninum* Nc-Spain1H isolate (MØ1H) *versus* non-infected macrophages (MØC). **Table S5.***Bos taurus* differentially expressed genes in bovine macrophages infected with *Neospora caninum* Nc-Spain7 isolate (MØ7) *versus* non-infected macrophages (MØC). **Table S6.***Bos taurus* differentially expressed genes in bovine macrophages inoculated with *Neospora caninum* heat-killed tachyzoites (MØHK) *versus* non-infected macrophages (MØC). **Table S7.***Bos taurus* differentially expressed genes in bovine macrophages infected with *Neospora caninum* Nc-Spain7 isolate (MØ7) *versus* those infected with Nc-Spain1H isolate (MØ1H).

**Additional file 5: Table S8.** Gene ontology analysis (Biological process) of differentially expressed genes in the comparison Nc-Spain1H-infected bovine macrophages *versus* non-infected macrophages**. Table S9.** Gene ontology analysis (Biological process) of differentially expressed genes in the comparison Nc-Spain7-infected bovine macrophages *versus* non-infected macrophages. **Table S10.** Gene ontology analysis (Biological process) of differentially expressed genes in the comparison bovine macrophages inoculated with heat-killed *Neospora caninum* tachyzoites *versus* non-infected macrophages.

**Additional file 6: Table S11.** Biological processes enriched from differentially expressed genes in MØ1H-MØC, MØ7-MØC, and MØHK-MØC comparisons.

**Additional file 7: Table S12**. KEGG pathway analysis of differentially expressed genes in the comparison Nc-Spain1H-infected bovine macrophages *versus* non-infected macrophages. **Table S13.** KEGG pathway analysis of differentially expressed genes in the comparison Nc-Spain7-infected bovine macrophages *versus* non-infected macrophages. **Table S14.** KEGG pathway analysis of differentially expressed genes in the comparison bovine macrophages inoculated with heat-killed *Neospora caninum* tachyzoites *versus* non-infected macrophages.

**Additional file 8: Figure S1.** Transcriptomic validation of RNA-seq analysis by RT-qPCR.

## Data Availability

All data analysed during this study are included in this published article and its additional files. Raw data are deposited in the NCBI Sequence Read Archive under the identifier PRJNA552526, https://www.ncbi.nlm.nih.gov/bioproject/PRJNA552526n.

## Abbreviations

ACTB: Actin beta
APC: Antigen presenting cell
BP: Biological process
BSA: Bovine serum albumin
BVDV: Bovine viral diarrhoea virus
CS: Condroitin sulfate
DAVID: Database for Annotation, Visualization and Integrated Discovery
DE: Differentially expressed
DEG: Differentially expressed genes
FC: Fold change
FOXO: Forkhead box O
GAG: Glycosaminoglycan
GAPDH: Glyceraldehyde-3-phosphate dehydrogenase
GO: Gene Ontology
HK: Heat-killed
Hpi: Hours post-infection
HS: Heparan sulfate
IBRV: Infectious bovine rhinotracheitis virus
KEGG: Kyoto Encyclopedia of Genes and Genomes
MHC: Major histocompatibility complex
MOI: Multiplicity of infection
MØ: Macrophage
MØC: Non-infected macrophages
MØHK: Macrophages inoculated with heat-killed tachyzoites
MØ1H: Macrophages inoculated with Nc-Spain1H
MØ7: Macrophages inoculated with Nc-Spain7
NK: Natural killer
NLR: NOD-like receptor
NOD: Nucleotide-binding and oligomerization domain
*P*adj: FDR-adjusted *P*-value
PBMC: Peripheral blood mononuclear cells
PRR: Pattern recognition receptor
RIN: RNA integrity number
ROS: Reactive oxygen species
RT: Room temperature
TLR: Toll-like receptor
