# Supplementary material for: Neospora caninum infection induces an isolate virulence-dependent pro-inflammatory gene expression profile in bovine monocyte-derived macrophages
Source: Parasit Vectors. 2020 Jul 25;13:374. doi: 10.1186/s13071-020-04239-3 (PMC7382829; doi:10.1186/s13071-020-04239-3)
Supplement: Supplementary file 1 — Additional file 1: Table S1. Sequences of primers used for transcriptomic validation by RT-qPCR. [file 13071_2020_4239_MOESM1_ESM.pdf]

Additional file 1: Table S1. Sequences of primers used for transcriptomic validation by RT-qPCR.

Ensembl id	Gene name	Gene symbol	Sequences (5' to 3')	Product size (bp)
ENSBTAG00000025257	C-C motif chemokine ligand 4	CCL4	AGCTGTGGTATTCCAGACCAA<br>TCAAGGTCATCCACGTACTCC[1]	87
ENSBTAG00000023659	Metallothionein-2	MT2	GGCTCCTGCAAATGCAAAGAT<br>CCGAAGCCCCTTTGCAGAC[2]	75
ENSBTAG00000021326	C-C motif chemokine ligand 20	CCL20	AATTAGCTGTGTGTGCAGATCC<br>CATCCTTTTGACTCTTTGACTGA[1]	80
ENSBTAG00000006894	Nitric oxide synthase, inducible	NOS2	GCAGCAGCGGCTCCATGAGG<br>CTTGGGCTGGTCAGGCAGGC[3]	141
ENSBTAG00000000436	TNF alpha induced protein 3	TNFAIP3	TGCTAAGCTGGCTGCAAGG<br>TTGCTGTGTTGATGCTGCG[4]	59
ENSBTAG00000021462	Colony stimulating factor 3	CSF3	CTTGGCCCTGCCCCGA<br>TTCCTCACTTGCTCTAAGCACTTG[4]	62
ENSBTAG00000001321	Interleukin 1 beta	IL1B	ACCTGAACCCATCAACGAAATG<br>TAGGGTCATCAGCCTCAAATAACA[1]	74
ENSBTAG00000006017	Nuclear factor kappa B subunit 2	NFKB2	CCTGCTGAATGCTCTGTCTG<br>TCCTCCTTCACCTCTGTGCT[1]	102
ENSBTAG00000010667	Phosphoinositide-3-kinase interacting protein 1	PIK3IP1	GGAGCTGGAATTGTCCTTGG<br>GCACACTTTCTGCTCGTGCT[1]	75
ENSBTAG00000046979	Legumain	LGMN	TGGAGGACCCTGAGGACG<br>TACCAGCCGTTTGATCCTGC[4]	64
ENSBTAG00000008008	Toll like receptor 2	TLR2	ACGACGCCTTTGTGTCCTAC<br>CCGAAAGCACAAAGATGGTT[5]	192
ENSBTAG00000008682	Toll like receptor 3	TLR3	GAGGCAGGTGTCCTTGAACT<br>GCTGAATTTCTGGACCCAAG[5]	329
ENSBTAG00000038149	NLR family pyrin domain containing 12	NLRP12	GCGGATTTTGTGGTTGAAGAT<br>CCTTCACACAGCAGCAGCAC[6]	106
ENSBTAG00000014921	Interleukin 6	IL6	CTGGGTTCAATCAGGCGATT<br>GGATCTGGATCAGTGTTCTGA[7]	150
ENSBTAG00000025471	Tumor necrosis factor	TNF	CCAGAGGGAAGAGCAGTCC<br>GGAGAGTTGATGTCGGCTAC[8]	126
ENSBTAG00000026199	Actin beta	ACTB	ACACCGCAACCAGTTCGCCAT<br>GTCAGGATGCCTCTCTTGCT[8]	216
ENSBTAG00000014731	Glyceraldehyde-3-phosphate dehydrogenase	GAPDH	ATCTCGCTCCTGGAAGATG<br>TCGGAGTGAACGGATTCG[9]	227

Ensembl id: Gene identifier in Ensembl database; bp: basepair

[1]Magee, D. A. *et al*. Global gene expression and systems biology analysis of bovine monocyte-derived macrophages in response to in vitro challenge with *Mycobacterium bovis*. *PLoS One* **7**, e32034 (2012).

[2]Fujie, T. *et al*. Induction of metallothionein isoforms by copper diethyldithiocarbamate in cultured vascular endothelial cells. *J. Toxicol. Sci.* **41**, 225-232 (2016).

[3]Li, R. W., Li, C. & Gasbarre, L. C. The vitamin D receptor and inducible nitric oxide synthase associated pathways in acquired resistance to *Cooperia oncophora* infection in cattle. *Vet. Res.* **42**, 48-9716-42-48 (2011).

[4]Chitko-McKown, C. G. *et al*. Gene expression profiling of bovine macrophages in response to *Escherichia coli* O157:H7 lipopolysaccharide. *Dev. Comp. Immunol.* **28**, 635-645 (2004).

[5]Menzies, M. & Ingham, A. Identification and expression of Toll-like receptors 1–10 in selected bovine and ovine tissues. *Vet. Immunol. Immunopathol.* **109**, 23-30 (2006).

[6]This study

[7]Arranz-Solís, D. *et al*. Systemic and local immune responses in sheep after *Neospora caninum* experimental infection at early, mid and late gestation. *Vet. Res.* **47**, 1-13 (2016).

[8]Regidor-Cerrillo, J. *et al*. *Neospora caninum* infection during early pregnancy in cattle: how the isolate influences infection dynamics, clinical outcome and peripheral and local immune responses. *Vet. Res.* **45**, 10-9716-45-10 (2014).

[9]Puech, C., Dedieu, L., Chantal, I. & Rodrigues, V. Design and evaluation of a unique SYBR Green real-time RT-PCR assay for quantification of five major cytokines in cattle, sheep and goats. *Vet. Res.* **11**, 1 (2015).
