# Supplementary material for: Neospora caninum infection induces an isolate virulence-dependent pro-inflammatory gene expression profile in bovine monocyte-derived macrophages
Source: Parasit Vectors. 2020 Jul 25;13:374. doi: 10.1186/s13071-020-04239-3 (PMC7382829; doi:10.1186/s13071-020-04239-3)
Supplement: Supplementary file 2 — Additional file 2: Table S2. Mapped and paired reads by sample against the Bos taurus genome. [file 13071_2020_4239_MOESM2_ESM.pdf]

Additional file 2: Table S2. Mapped and paired reads by sample against *Bos taurus* genome.

Sample	Total reads	Mapped reads	% mapped reads	High quality reads	% high quality reads	Splice reads	% splice reads
**MØC R1**	57978010	57066703	98.43	39294793	67.78	12982893	22.39
**MØC R2**	48884602	48177970	98.55	33370638	68.26	10548504	21.58
**MØC R3**	49634030	48097516	96.90	32464469	65.41	9432877	19.00
**MØ1H R1**	45726510	38902639	85.08	26406712	57.75	7926380	17.33
**MØ1H R2**	49810014	45221494	90.79	29675634	59.58	9187373	18.44
**MØ1H R3**	46709092	42000811	89.92	30077799	64.39	9428874	20.19
**MØ7 R1**	50818888	42614504	83.86	26894321	52.92	8298603	16.33
**MØ7 R2**	59129224	51174478	86.55	32684186	55.28	10153185	17.17
**MØ7 R3**	48795612	39459440	80.87	28423812	58.25	8812206	18.06
**MØHK R1**	45889532	45436847	99.01	31928506	69.58	10123030	22.06
**MØHK R2**	36301526	36482426	100.00	16726768	46.08	5510135	15.18
**MØHK R3**	51781248	48520326	93.70	32549263	62.86	10305755	19.90

MØC: Non-infected macrophages; MØ1H: macrophages inoculated with Nc-Spain1H; MØ7: macrophages inoculated with Nc-Spain7; MØHK: macrophages inoculated with *N. caninum* heat-killed tachyzoites; R1-R3: Biological replicates 1-3 collected from three independent experiments.
