# Supplementary material for: Neospora caninum infection induces an isolate virulence-dependent pro-inflammatory gene expression profile in bovine monocyte-derived macrophages
Source: Parasit Vectors. 2020 Jul 25;13:374. doi: 10.1186/s13071-020-04239-3 (PMC7382829; doi:10.1186/s13071-020-04239-3)
Supplement: Supplementary file 6 — Additional file 6: Table S11. Biological processes enriched from differentially expressed genes in MØ1H-MØC, MØ7-MØC, and MØHK-MØC comparisons. [file 13071_2020_4239_MOESM6_ESM.pdf]

Additional file 6: Table S11. Biological processes enriched from differentially expressed genes in MØ1H-MØC, MØ7-MØC, and MØHK-MØC comparisons.

MØ1H *versus* MØC						
GO term ID	Description	padj	Count	Frequency [a]	Uniqueness [b]	Dispensability [c]
GO:0002376	**Immune system process**	5.16E-04	128	10.27 %	0.97	0.00
GO:0002684	**PR of immune system process**	3.01E-02	60	3.65 %	0.72	0.00
GO:0051249	*Regulation of lymphocyte activation*	2.07E-02	25	1.77 %	0.73	0.83
GO:0006955	*Immune response*	2.39E-05	88	6.05 %	0.75	0.67
[d]GO:0045321	*Leukocyte activation*	1.36E-02	41	3.51 %	0.77	0.61
GO:0002521	*Leukocyte differentiation*	4.16E-03	33	2.47 %	0.76	0.74
[d]GO:1903708	*PR of hemopoiesis*	2.88E-03	41	0.80 %	0.70	0.69
GO:0006732	**Coenzime metabolic process**	1.67E-02	33	1.38 %	0.94	0.00
GO:0006954	**Inflammatory response**	5.74E-05	58	2.67 %	0.87	0.00
GO:0050729	*PR of inflammatory response*	5.74E-05	18	0.41 %	0.76	0.76
GO:0031349	*PR of defense response*	1.35E-04	32	1.29 %	0.77	0.66
GO:0007159	**Leukocyte cell-cell adhesion**	1.23E-03	35	2.37 %	0.88	0.00
[d]GO:0046634	*Regulation of alfa-beta T cell-activation*	9.40E-03	10	0.33 %	0.72	0.69
GO:1903039	*PR of leukocyte cell-cell adhesion*	1.02E-02	18	0.82 %	0.81	0.82
[d]GO:0002286	*T cell activation involved in immune response*	1.36E-03	12	0.57 %	0.72	0.73
[d]GO:0016032	**Viral process**	3.20E-02	43	1.24 %	0.91	0.00
[d]GO:0044419	*Interspecies interaction between organisms*	3.92E-02	44	1.46 %	0.92	0.65
[d]GO:0044403	*Symbiosis, mutualism through parasitism*	3.92E-02	44	1.44 %	0.91	0.96
GO:0050663	**Cytokine secretion**	1.23E-03	20	0.85 %	0.78	0.02
[d]GO:0042990	*Regulation of TF import into nucleus*	1.40E-02	10	0.40 %	0.78	0.68
[d]GO:0042991	*TF import into nucleus*	1.40E-02	10	0.41 %	0.83	0.78
GO:0001817	*Regulation of cytokine production*	1.12E-02	38	3.51 %	0.83	0.81
[d]GO:0070201	*Regulation of protein localization*	1.40E-02	50	3.44 %	0.78	0.55
GO:0046822	*Regulation of nucleocytoplasmic transport*	3.93E-02	17	0.98 %	0.80	0.75
[d]GO:0051046	*Regulation of secretion*	4.45E-03	46	2.86 %	0.78	0.72
GO:0050707	*Regulation of cytokine secretion*	2.87E-03	16	0.72 %	0.73	0.79
GO:0032943	**Mononuclear cell proliferation**	1.92E-02	21	1.42 %	0.89	0.02
GO:0032944	*Regulation of mononuclear cell proliferation*	4.59E-02	17	0.93 %	0.85	0.93
GO:0043067	**Regulation of programmed cell death**	1.19E-03	87	6.47 %	0.88	0.09
GO:0050793	**Regulation of developmental process**	3.92E-02	107	10.36 %	0.89	0.09
[d]GO:0051707	**Response to other organism**	1.23E-03	61	3.57 %	0.87	0.14
[d]GO:0043207	*Response to external biotic stimulus*	1.23E-03	61	3.58 %	0.87	0.95
GO:0043408	**Regulation of MAPK cascade**	1.80E-03	52	2.97 %	0.65	0.14
GO:1902531	*Regulation of intracellular signal transduction*	2.20E-04	104	7.58 %	0.74	0.62
GO:0023057	*NR of signaling*	9.40E-03	70	5.32 %	0.85	0.62
GO:0042325	*Regulation of phosphorylation*	6.28E-03	87	6.87 %	0.82	0.75
GO:0016310	*Phosphorylation*	1.22E-02	128	11.27 %	0.89	0.55
GO:0010648	*NR of cell communication*	9.40E-03	70	5.30 %	0.84	0.63
GO:0006468	*Protein phosphorylation*	2.00E-02	101	9.30 %	0.87	0.77
[d]GO:0010647	*PR of cell communication*	4.94E-02	80	6.61 %	0.81	0.65
GO:0071345	**Cellular response to cytokine stimulus**	3.80E-03	41	2.93 %	0.88	0.18
GO:0034097	*Response to cytokine*	1.11E-02	44	3.27 %	0.88	0.55
GO:0006509	**Membrane protein ectodomain proteolysis**	1.55E-02	8	0.25 %	0.94	0.18
GO:0001816	**Cytokine production**	1.23E-02	43	3.03 %	0.91	0.23

GO:0098869	**Cellular oxidant detoxification**	4.46E-02	13	0.46 %	0.90	0.28
GO:0009636	**Response to toxic substance**	2.81E-02	16	0.73 %	0.90	0.30
GO:0010883	**Regulation of lipid storage**	3.92E-02	8	0.20 %	0.84	0.33
GO:0006082	**Organic acid metabolic process**	3.74E-03	82	4.72 %	0.86	0.38
GO:0044712	**Single-organism catabolic process**	2.81E-02	66	3.68 %	0.87	0.39
[d]GO:1901575	*Organic substance catabolic process*	2.20E-02	127	8.73 %	0.92	0.76
GO:0050790	**Regulation of catalytic activity**	2.27E-02	124	10.94 %	0.88	0.46
GO:0044093	*PR of molecular function*	2.81E-02	90	7.90 %	0.89	0.72
GO:0048585	**NR of response to stimulus**	3.80E-03	83	6.25 %	0.79	0.49
[d]GO:0006952	**Defense response**	5.74E-05	98	5.99 %	0.87	0.50
MØ7 *versus* MØC						
GO term ID	Description	padj	Count	Frequency [a]	Uniqueness [b]	Dispensability [c]
GO:0002376	**Immune system process**	6.98E-04	109	10.27 %	0.97	0.00
GO:0007159	**Leukocyte cell-cell adhesion**	2.54E-03	30	2.37 %	0.89	0.00
[e]GO:0042110	*T cell activation*	1.49E-02	25	2.18 %	0.73	0.86
GO:1903039	*PR of leukocyte cell-cell adhesion*	4.79E-02	14	0.82 %	0.82	0.76
[e]GO:0022610	**Biological adhesion**	1.67E-02	68	6.66 %	0.97	0.00
GO:0031349	**PR of defense response**	9.10E-06	31	1.29 %	0.77	0.00
GO:0050729	*PR of inflammatory response*	5.69E-06	18	0.41 %	0.79	0.76
GO:0006954	*Inflammatory response*	7.59E-05	51	2.67 %	0.86	0.66
[e]GO:0070661	**Leukocyte proliferation**	1.52E-02	20	1.48 %	0.89	0.02
GO:0001816	**Cytokine production**	4.20E-03	40	3.03 %	0.90	0.02
GO:0006082	**Organic acid metabolic process**	1.14E-02	68	7.72 %	0.86	0.03
GO:0050790	**Regulation of catalytic activity**	1.12E-02	109	10.94 %	0.87	0.05
[e]GO:0044092	*NR of molecular function*	4.51E-02	54	5.20 %	0.87	0.66
GO:0044093	*PR of molecular function*	3.75E-02	76	7.90 %	0.87	0.72
GO:0006509	**Membrane protein ectodomain proteolysis**	2.57E-02	7	0.25 %	0.93	0.05
GO:0006732	**Coenzyme metabolic process**	1.69E-02	29	1.38 %	0.94	0.08
GO:0042981	**Regulation of apoptotic process**	4.70E-04	76	6.08 %	0.86	0.11
GO:0071345	**Cellular response to cytokine stimulus**	4.52E-03	36	2.93 %	0.87	0.12
GO:0034097	*Response to cytokine*	1.00E-02	39	3.27 %	0.88	0.55
[e]GO:0009605	**Response to external stimulus**	1.13E-03	103	8.74 %	0.90	0.16
[e]GO:0014033	**Neural crest cell differentiation**	3.74E-02	7	0.34 %	0.86	0.21
GO:0050707	**Regulation of cytokine secretion**	5.03E-03	14	0.72 %	0.70	0.23
GO:0050663	*Cytokine secretion*	3.63E-03	17	0.85 %	0.76	0.97
[e]GO:1903531	*NR of secretion by cell*	5.64E-03	17	0.69 %	0.74	0.71
GO:0001817	*Regulation of cytokine production*	5.17E-03	35	2.72 %	0.81	0.81
[e]GO:1904589	*Regulation of protein import*	6.64E-03	16	0.81 %	0.78	0.85
[e]GO:1900180	*Regulation of protein localization to nucleus*	1.18E-02	18	1.04 %	0.78	0.85
GO:0046822	*Regulation of nucleocytoplasmic transport*	1.14E-02	17	0.98 %	0.80	0.81
[e]GO:0042306	*Regulation of protein import into nucleus*	5.17E-03	16	0.80 %	0.76	0.69
GO:0050793	**Regulation of developmental process**	3.07E-02	92	10.36 %	0.86	0.25
[e]GO:0046470	**Phosphatidylcholine metabolic process**	3.75E-02	6	0.18 %	0.86	0.27
GO:0002684	**PR of immune system process**	1.01E-03	52	3.65 %	0.74	0.27
[e]GO:1902107	*PR of leukocyte differentiation*	1.89E-02	13	0.64 %	0.69	0.72
GO:0032943	*Mononuclear cell proliferation*	1.52E-02	19	1.42 %	0.66	0.97
GO:0032944	*Regulation of mononuclear cell proliferation*	4.68E-02	15	0.93 %	0.82	0.94
GO:0051249	*Regulation of lymphocyte activation*	3.83E-02	21	1.77 %	0.74	0.81
GO:0006955	*Immune response*	5.69E-06	78	6.05 %	0.77	0.67

[e]GO:0046651	*Lymphocyte proliferation*	1.52E-02	19	1.41 %	0.71	0.54
GO:0002521	*Leukocyte differentiation*	1.04E-02	28	2.47 %	0.74	0.58
GO:0098869	**Cellular oxidant detoxification**	3.26E-02	12	0.46 %	0.90	0.28
GO:0009636	**Response to toxic substance**	1.69E-02	15	0.73 %	0.90	0.30
GO:0010883	**Regulation of lipid storage**	1.74E-02	8	0.20 %	0.84	0.34
GO:0009611	**Response to wounding**	4.94E-02	26	1.96 %	0.88	0.38
GO:0044712	**Single-organism catabolic process**	4.80E-02	55	3.68 %	0.90	0.39
GO:0043408	**Regulation of MAPK cascade**	2.37E-04	49	2.97 %	0.63	0.40
GO:1902531	*Regulation of intracellular signal transduction*	2.37E-04	90	7.58 %	0.73	0.62
GO:0023057	*NR of signaling*	4.05E-03	63	5.32 %	0.81	0.62
GO:0051248	*NR of protein metabolic process*	2.19E-02	58	5.29 %	0.81	0.56
GO:0042325	*Regulation of phosphorylation*	1.49E-02	73	6.87 %	0.80	0.75
GO:0010648	*NR of cell communication*	4.05E-03	63	5.30 %	0.80	0.63
GO:0006468	*Protein phosphorylation*	2.95E-02	85	9.30 %	0.85	0.77
GO:0048585	**NR of response to stimulus**	9.28E-04	75	6.25 %	0.76	0.49
	MØHK *versus* MØC					
GO term ID	Description	padj	Count	Frequency [a]	Uniqueness [b]	Dispensability [c]
GO:0002376	**Immune system process**	2,01E-02	62	10.27 %	0.96	0.00
GO:0006954	**Inflammatory response**	8,37E-04	34	2.67 %	0.78	0.00
GO:0050729	*PR of inflammatory response*	9,35E-04	12	0.41 %	0.69	0.58
GO:0031349	*PR of defense response*	3,17E-02	15	1.29 %	0.70	0.76
[f]GO:0032103	*PR of response to external stimulus*	2,03E-02	15	1.15 %	0.72	0.68
GO:0009611	*Response to wounding*	3,28E-03	23	1.96 %	0.81	0.42
[f]GO:0007155	**Cell adhesion**	2,91E-02	42	6.62 %	0.96	0.00
[f]GO:0040011	**Locomotion**	3,22E-02	42	7.12 %	0.96	0.00
[f]GO:0050817	**Coagulation**	9,93E-03	15	0.92 %	0.92	0.00
[f]GO:0050878	**Regulation of body fluid levels**	5,15E-03	22	1.52 %	0.90	0.00
GO:0050900	**Leukocyte migration**	2,25E-02	16	1.42 %	0.82	0.02
GO:0006955	*Immune response*	2,77E-02	39	6.05 %	0.81	0.58
[f]GO:0030335	*PR of cell migration*	3,59E-02	17	1.82 %	0.76	0.66
[f]GO:0008283	**Cell proliferation**	5,66E-03	54	8.54 %	0.94	0.02
GO:0048660	**Regulation of smooth muscle cell proliferation**	2,91E-02	7	0.33%	0.87	0.04
GO:0048659	*Smooth muscle cell proliferation*	2,91E-02	7	0.34 %	0.91	0.90
[f]GO:0051348	**NR of transferase activity**	1,69E-02	17	1.40 %	0.87	0.04
[f]GO:0043547	*PR of GTPase activity*	3,62E-02	19	2.67 %	0.88	0.50
[f]GO:0006928	**Movement of cell or subcellular component**	3,68E-02	45	7.97 %	0.92	0.10
GO:0043408	**Regulation of MAPK cascade**	9,93E-03	29	2.97 %	0.55	0.14
GO:0016310	*Phosphorylation*	3,36E-02	65	11.27 %	0.83	0.68
GO:0048585	*NR of response to stimulus*	9,62E-03	46	6.26 %	0.67	0.49
[f]GO:0080134	**Regulation of response to stress**	4,31E-02	38	5.11 %	0.71	0.54
GO:1902531	*Regulation of intracellular signal transduction*	1,09E-03	57	7.58 %	0.67	0.62
[f]GO:0033673	*NR of kinase activity*	1,69E-02	16	1.29 %	0.68	0.88
GO:0006468	*Protein phosphorylation*	4,31E-02	52	9.30 %	0.81	0.66
GO:0023057	*NR of signaling*	2,94E-02	37	5.32 %	0.77	0.62
GO:0010648	*NR of cell communication*	2,94E-02	37	5.30 %	0.78	0.63
[f]GO:0045936	*NR of phosphate metabolic process*	1,69E-02	24	2.70 %	0.72	0.65
GO:0051248	*NR of protein metabolic process*	2,91E-02	37	5.29 %	0.76	0.56
[f]GO:0010563	*NR of phosphorus metabolic process*	1,69E-02	24	2.70 %	0.73	0.65

GO:0034097	**Response to cytokine**	1,69E-02	26	3.27 %	0.79	0.14
[f]GO:0002237	*Response to molecule of bacterial origin*	4,31E-02	14	1.30 %	0.77	0.49
[f]GO:0034612	*Response to tumor necrosis factor*	1,69E-02	10	0.81 %	0.80	0.47
GO:0071345	*Cellular response to cytokine stimulus*	1,70E-02	23	2.93 %	0.77	0.79
GO:0050707	**Regulation of cytokine secretion**	1,69E-02	10	0.72 %	0.79	0.20
GO:0050663	*Cytokine secretion*	4,62E-02	10	0.852%	0.84	0.79
[f]GO:0051049	*Regulation of transport*	3,36E-02	50	7.79 %	0.81	0.51
GO:0098869	**Cellular oxidant detoxification**	3,46E-02	9	0.46 %	0.84	0.28
[f]GO:0006694	**Steroid biosynthetic process**	3,95E-02	10	0.66 %	0.89	0.29
[f]GO:0044283	*Small molecule biosynthetic process*	4,31E-02	26	2.35 %	0.89	0.59
GO:0009636	**Response to toxic substance**	2,22E-02	11	0.73 %	0.83	0.30

[a]Proportion of the GO term in the underlying protein annotation database (UniProt). Higher frequency implies more general terms and lower frequency more specific ones.

[b]Measures whether the term is an outlier when compared semantically to the whole list. Calculated as 1- (average semantic similarity of a term to all other terms). More unique terms tend to be less dispensable.

[c]Semantic similarity threshold at which the term was removed from the list and assigned to a cluster.

[d]GO terms not enriched from DEG in MØ7-MØC or MØHK-MØC comparisons.

[e]GO terms not enriched from DEG in MØ1H-MØC or MØHK-MØC comparisons.

[f]GO terms not enriched from DEG in MØ7-MØC or MØ1H-MØC comparisons.

MØ1H, macrophages inoculated with Nc-Spain1H; MØ7, macrophages inoculated with Nc-Spain7; MØHK, macrophages inoculated with heat-killed tachyzoites; MØC, non-infected macrophages; PR, positive regulation; NR negative regulation; TF transcription factor. Representative GO terms for each of the clusters are shown in bold and GO terms included within the clusters are italicized.
