# Supplementary material for: Neospora caninum infection induces an isolate virulence-dependent pro-inflammatory gene expression profile in bovine monocyte-derived macrophages
Source: Parasit Vectors. 2020 Jul 25;13:374. doi: 10.1186/s13071-020-04239-3 (PMC7382829; doi:10.1186/s13071-020-04239-3)
Supplement: Supplementary file 8 — Additional file 8: Figure S1. Transcriptomic validation of RNA-seq analysis by RT-qPCR. [file 13071_2020_4239_MOESM8_ESM.pdf]

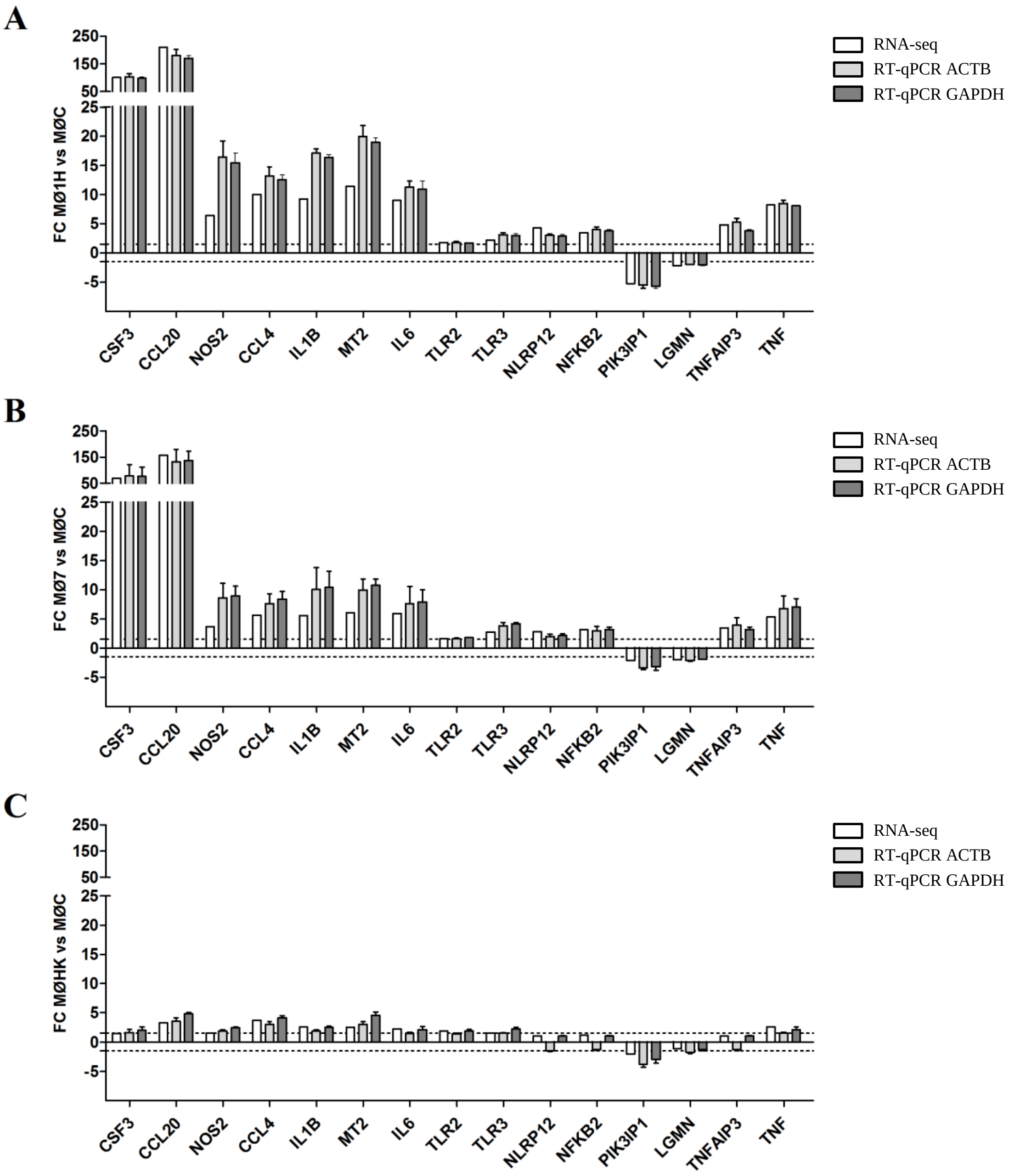

Additional file 8: Figure S1. Transcriptomic validation of RNA-seq analysis by RT-qPCR. Fold changes (FC) in gene expression of *Bos taurus* genes in the comparisons between (A) Nc-Spain1H-infected macrophages (MØ1H) *versus* non-infected macrophages (MØC), (B) Nc-Spain7-infected macrophages (MØ7) *versus* MØC, and (C) macrophages inoculated with heat-killed *N. caninum* tachyzoites (MØHK) *versus* MØC. Gene expression was measured by quantitative real-time PCR (RT-qPCR) and normalized with housekeeping Actin beta (ACTB) and Glyceralde-3-phosphate dehydrogenase (GAPDH). All the differentially expressed genes (FC ≥ 1.5, delimited by a discontinuous line) showed similar expression profile for both techniques and similar FC for the two housekeepings. Lack of correlation was only observed for three genes expressed by MØHK with FC < 1.5 and normalized with ACTB.
